# Prehospital triage tools across the world: a scoping review of the published literature

**DOI:** 10.1186/s13049-022-01019-z

**Published:** 2022-04-27

**Authors:** Smitha Bhaumik, Merhej Hannun, Chelsea Dymond, Kristen DeSanto, Whitney Barrett, Lee A. Wallis, Nee-Kofi Mould-Millman

**Affiliations:** 1 Department of Emergency Medicine, Denver Health and Hospital Authority, 777 Bannock St, Denver, CO 80204 USA; 2 Department of Emergency Medicine, School of Medicine, University of Colorado, 12631 E. 17th Ave, Room 2612, MS C326, Aurora, CO 80045 USA; 3 Department of Family Medicine, Reading Hospital – Tower Health, 420 South 5th Avenue, West Reading, PA 19611 USA; 4 Department of Emergency Medicine, Providence St Joseph Hospital, 2700 Dolbeer St, Eureka, CA 95501 USA; 5 Strauss Health Sciences Library, School of Medicine, University of Colorado Anschutz Medical Campus, 12950 E. Montview Blvd., Mail Stop A003, Aurora, CO 80045 USA; 6 Department of Emergency Medicine, University of New Mexico Health Sciences Center, 1 University of New Mexico, MSC11 6025, Albuquerque, NM 87131 USA; 7 Division of Emergency Medicine, Groote Schuur Hospital, University of Cape Town, F51 Old Main Building, Observatory, Cape Town, 7935 South Africa

**Keywords:** Prehospital, Triage, Trauma, Stroke, Emergency medical services, EMS, Tools, Scoping review, Global health, International

## Abstract

**Background:**

Accurate triage of the undifferentiated patient is a critical task in prehospital emergency care. However, there is a paucity of literature synthesizing currently available prehospital triage tools. This scoping review aims to identify published tools used for prehospital triage globally and describe their performance characteristics.

**Methods:**

A comprehensive search was performed of primary literature in English-language journals from 2009 to 2019. Papers included focused on emergency medical services (EMS) triage of single patients. Two blinded reviewers and a third adjudicator performed independent title and abstract screening and subsequent full-text reviews.

**Results:**

Of 1521 unique articles, 55 (3.6%) were included in the final synthesis. The majority of prehospital triage tools focused on stroke (n = 19; 35%), trauma (19; 35%), and general undifferentiated patients (15; 27%). All studies were performed in high income countries, with the majority in North America (23, 42%) and Europe (22, 40%). 4 (7%) articles focused on the pediatric population. General triage tools aggregate prehospital vital signs, mental status assessments, history, exam, and anticipated resource need, to categorize patients by level of acuity. Studies assessed the tools’ ability to accurately predict emergency department triage assignment, hospitalization and short-term mortality. Stroke triage tools promote rapid identification of patients with acute large vessel occlusion ischemic stroke to trigger timely transport to diagnostically- and therapeutically-capable hospitals. Studies evaluated tools’ diagnostic performance, impact on tissue plasminogen activator administration rates, and correlation with in-hospital stroke scales. Trauma triage tools identify patients that require immediate transport to trauma centers with emergency surgery capability. Studies evaluated tools’ prediction of trauma center need, under-triage and over-triage rates for major trauma, and survival to discharge.

**Conclusions:**

The published literature on prehospital triage tools predominantly derive from high-income health systems and mostly focus on adult stroke and trauma populations. Most studies sought to further simplify existing triage tools without sacrificing triage accuracy, or assessed the predictive capability of the triage tool. There was no clear ‘gold-standard’ singular prehospital triage tool for acute undifferentiated patients.

***Trial registration*:**

Not applicable.

**Supplementary Information:**

The online version contains supplementary material available at 10.1186/s13049-022-01019-z.

## Background

Emergency medical services (EMS) systems deliver prehospital care and transport to victims of sudden illness or injury [1]. A critical task in prehospital emergency care is the accurate triage of the undifferentiated patient which helps dictate the ensuing treatment and/or transportation plan. Triage has a demonstrated mortality benefit, for example, in the setting of ST-segment elevation myocardial infarction (STEMI), stroke and trauma [2–4]. Triage is employed repeatedly and used across the spectrum of emergency care delivery: at the time of resource dispatch, upon prehospital personnel arrival at the scene of the patient, and again by staff of the receiving facility [5].

In low- and middle-income countries (LMICs) in particular, prehospital triage may play an even more critical role. It is estimated that more than half of deaths in LMICs are caused by conditions that benefit from prehospital and emergency care. Examples include infectious diseases, complications of pregnancy, cardiovascular disease, road traffic accidents and interpersonal violence [6]. Many LMICs have nascent EMS systems that would benefit from effective triage tools [7–9].

As EMS systems develop globally, regardless of country or setting, there is a general paucity of review literature appraising prehospital triage tools currently in use across the world. The only comprehensive search of prehospital triage tools to date is a 2013 systematic review which assessed patient-level outcomes attributable to validated prehospital triage systems [10]. Despite screening over 11,000 unique titles and abstracts, and performing 120 full text reviews, the authors found no studies that met their inclusion criteria, which required the triage tool to undergo direct comparison to an alternate tool or to a no triage arm. Hence, more exploratory studies are needed to better understand the state of prehospital triage across the globe in an effort to inform future EMS research and development.

The primary aim of this scoping review was to identify the breadth and diversity of published prehospital triage tools in use across the world and to understand reasons why these studies were performed. Secondarily, we sought to describe the performance characteristics of these tools to provide recommendations on which tools, if any, may be suitable for adoption in new and developing EMS systems.

## Methods

A scoping review was done to systematically map the research on prehospital triage tool development, and to identify any existing gaps in knowledge [11]. The following research questions were formulated: What is known from the literature about triage tools that are being used by EMS providers at the time of single patient care in the prehospital setting globally? How are these tools being studied, and what are their performance characteristics?

Considering our focus on triage in routine EMS care, we did not include mass casualty triage tools given their unique mode of application to sorting patients in the specific circumstance of multi-casualty events. We defined prehospital triage as the algorithmic process undertaken by an EMS provider to sort the undifferentiated patient into an appropriate category based on suspected pathology and level of acuity. Clinical treatment protocols (e.g., step-by-step prehospital asthma treatment), clinical guidelines, and singular technology-dependent triage tools (e.g., electrocardiogram (EKG) for prehospital STEMI triage) are excluded from our definition of triage.

A medical librarian performed a comprehensive literature search in December 2019. Relevant publications were identified by searching a combination of index terms and keywords for the concepts of triage and pre-hospital care in the following databases: MEDLINE (via Ovid MEDLINE® and Epub Ahead of Print, In-Process and Other Non-Indexed Citations, Daily and Versions®, 1946 to present), Embase (via Elsevier, Embase.com, 1947 to present), and Web of Science Core Collection (via Clarivate Analytics, including Science Citation Index Expanded 1974 to present, and Social Sciences Citation Index 1974 to present).

Results were limited to English language articles published between 2009 and 2019 to include more contemporaneous papers that are more likely to study tools in current use. We reviewed scientifically peer-reviewed published literature; publication types were limited to the primary literature, including observational cohort and interventional studies. Since we sought to perform a direct review of the most robust primary literature studying these tools, we excluded case reports, reviews, systematic reviews, meta-analyses, comments, editorials, letters, and conference proceedings [12]. All results were exported to, and deduplicated in, EndNote X9 (Clarivate Analytics, Philadelphia, PA). Covidence systematic review software (Veritas Health Innovation, Melbourne, Australia) was used for screening and full text review. See Additional file 1 for a list of all database search strategies.

Retrieved articles were independently screened by two trained reviewers (A1, A2), blinded to each other’s reviews. During screening, each reviewer read article titles and abstracts to determine if they satisfied inclusion criteria, and to ensure they did not meet any exclusion criteria (see Table 1). Articles were scored as ‘yes’, ‘no’, or ‘maybe’. Discrepant reviews, or any reviews marked as ‘maybe’, were adjudicated by a third reviewer (A3).

**Table 1:** Article inclusion and exclusion criteria

Inclusion criteria	Exclusion criteria
Prehospital/EMS focused*	In-hospital focus only
In-depth description of triage tool included^#^	Hypothetical triage tool^@^
Triage tool/process must be a main focus^$^	Systematic review/meta-analysis
Traditional ground and aeromedical EMS system	Atypical EMS systems
Observational studies with n ≥ 50	Observational study with n < 50
Interventional studies	Triage by EMS dispatch/communications center
	Mass casualty triage tool
	Termination of resuscitation tool
	Prehospital clinical algorithm or protocol^^^

*The study had to specifically include patient-level prehospital data

^#^The triage tool must be fully described within the article or through a provided reference. The tool should help the provider arrive at a specific, often binary, triage decision (e.g., Transport patient to trauma center or not; label patient as low or high acuity)

^$^Assessment of triage outcomes or process must be a stated primary or secondary objective of the study

^@^The triage tool is not actively used in prehospital clinical practice, is used for research purposes only, or is in development

^Excludes EMS agency prehospital algorithms or protocols used for clinical management en route (e.g., “asthma protocol”) or those that rely on a single diagnostic tool such as a fingerstick glucose or EKG to make a triage decision (e.g., “chest pain protocol”)

The final list of included (‘yes’) articles was divided between the two reviewers (A1, A2) for a full text review and critical synthesis. The full manuscript of each article was reviewed in detail, and if an article was deemed to meet one or more exclusion criteria, then it was excluded with reason(s) provided. Full text review articles were summarized in prose in a paragraph format (see Additional file 2) which note findings of most relevance to the research objectives. Data from articles, including tool name, country, population, primary research question, sample size, and major findings were also coded in a data charting summary table (See Table 2 for a summary of the top one-third highest quality studies and Additional file 3 for a summary of all studies). The investigators independently appraised, then collectively discussed, all major findings through independent review of the summary paragraphs and tables to reach consensus regarding major themes, key conclusions and recommendations.

**Table 2:** Performance characteristics of prehospital triage tools from a subset of the highest quality articles

Type	Study	Tool name	Pop	Primary research question	Major findings
General	Meisel 2009	PEAR	A	To validate PEAR for predicting hospital admission using routinely collected out-of-hospital information	AUC for combined cohort was 0.83 for all admissions and 0.72 for ICU admissions. n = 1102
	Hoikka 2018	NEWS	All	To examine the accuracy of the prehospitally implemented NEWS in predicting 1-day and 30-day mortalities in an unselected EMS population	The high-risk NEWS group (score ≥ 7) had sensitivities for 1-day and 30-day mortalities of 0.801 (CI 0.74–0.86) and 0.42 (CI 0.38–0.47), respectively. n = 12,426
	Leeies 2017	CTAS	A	To prospectively evaluate CTAS interrater reliability between EMS providers and ED triage nurses	Interrater reliability κw = 0.437 (*p* < 0.001, 95% CI 0.421–0.452). n = 14,378
	Magnusson 2019	RETTS-p	P	To evaluate agreement between the EMS field assessment using RETTS-p and the hospital diagnosis of emergent condition	Sn 66.7% and Sp 67.0%, with under-triage rate 33%, over-triage rate 33.3%. n = 716
Stroke	Helwig 2019	LAMS	A	To compare LAMS to Mobile Stroke Unit (MSU) in accurately triaging patients to the appropriate stroke hospital (CSC vs PSC)	An accurate triage decision was reached for 69.8% in the LAMS group and 100% in the MSU group (difference, 30.2%; 95% CI, 17.8%-42.5%; P < 0.001). n = 116
	Carrera 2019	RACE	A	To revalidate RACE after its region-wide implementation in Catalonia	RACE ≥ 5 showed Sn 84%, Sp 60%, AUC 0.77, for detecting LVO. N = 1822
	Jumaa 2019	RACE	A	To report performance characteristics of RACE for LVO eligible for mechanical thrombectomy	A RACE cut-off point of ≥ 5 had Sn 77%, Sp 75%, PPV 0.97, NPV 0.25, accuracy 75.3% (95%CI 73.1–77.4). n = 1147
Trauma triage	Brown 2011	FTDS	A	To analyze whether trauma center need was accurately predicted solely by the physiologic (PHY) and anatomic (ANA) criteria of the FTDS	Application of only the PHY and ANA criteria identifies trauma center need with Sn 49%, Sp 78% and undertriage rate of 51%. Mechanism of injury and special considerations criteria play an important role in minimizing under-triage rates. n = 1,086,764
	Newgard 2011	FTDS	All	To evaluate diagnostic performance of FTDS for identifying major trauma (Injury Severity Score [ISS] ≥ 16)	Sn 85.8% (95% CI 85.0–86.6) and Sp 68.7% (95% CI 68.4–68.9). n = 122,345
	Barnett 2013	FTDS	All	To describe the use of field triage criteria by EMS personnel in the Western United States	The three most common criteria cited (of 33 in use) were EMS provider judgment, age < 5 or > 55 years, and GCS < 14. n = 46,414
	Davidson 2014	FTDS	All	To determine the likelihood of serious trauma based on vehicle damage sustained in a crash as described in Step 3 of the FTDS	Crash characteristics that predict severe injury included intrusion of greater than 12 inches (PPV of 10.4%; 95% CI, 9.5–11.3) and steering wheel collapse (PPV of 25.7%; 95% CI, 23.0–28.4%). n = 85,761
	Lerner 2017	FTDS	P	To determine the change in under- and over-triage rates when the 2011 Field Triage Guidelines are compared to the 2006 and 1999 versions	Applying the 1999, 2006, or 2011 Guidelines to the EMS interview data the over-triage rate was 32.6%, 27.9%, and 28.0%, respectively. The under-triage rate was 26.5%, 35.1%, and 34.8%, respectively. The 2011 Guidelines resulted in an 8.2% (95% CI 0.6–15.9%) absolute increase in under-triage and a 4.6% (95% CI 2.8–6.3%) decrease in over-triage compared to 1999 Guidelines. n = 5594
	Ardolino 2015	PTS	P	To assess performance of English pediatric prehospital trauma triage tools	East Midlands (18%), North West (21%) and Northern (19%) tools had the best over-triage rates. All had under-triage rates of 0%. n = 2934
	Cox 2012	VSTTC	A	To evaluate performance of the Victorian prehospital trauma triage criteria in discriminating for major trauma	Sn 95.3%, Sp 62.7%, under-triage rate 4.7%, and over-triage rate 37.3% for major trauma. n = 45,332
	vanLaarhoven 2014	Dutch	A	To evaluate the protocol's ability to identify severely injured adult trauma patients (ISS > = 16)	Sn 89.1%, Sp 60.5%, PPV 26.5%, NPV 97.2%, undertriage rate 10.9%, overtriage rate 39.5%. n = 1607
Trauma for helicopter EMS	Brown 2012	FTDS	All	To determine which FTDS criteria can be used by field EMS providers to predict which trauma patients would benefit from helicopter transport	Odds of increased survival to discharge by helicopter transport found in following conditions: GCS < 14 (aOR 1.22); respiratory rate < 10 or > 29 (aOR 1.32); penetrating injury (aOR 1.40), age > 55 (aoR 1.15). n = 258,387
	Brown 2017	AMPT	A	To validate the effectiveness of the AMPT score to identify patients with a survival benefit from helicopter EMS (HEMS)	For AMPT score ≥ 2, HEMS increases odds of in-hospital survival by 6.7% (ARR 1.067; 95% CI 1.040–1.083, *p* < 0.001). n = 222,827
Traumatic brain injury	Fuller 2016	HITS-NS	A	To determine the accuracy of the HITS-NS triage rule for identifying patients with significant TBI	Sn 28.3% and Sp 94.4% for significant TBI. n = 3828

PEAR, Philadelphia EMS Admission Rule; NEWS, National Early Warning Score; CTAS, Canadian Triage and Acuity Scale; RETTS-p, Rapid Emergency Triage and Treatment System-pediatrics; LAMS, Los Angeles Motor Scale; RACE, Rapid Arterial Occlusion Evaluation; FTDS, Field Triage Decision Scheme; PTS, Pediatric trauma score, pediatric triage tape, East Midlands, London, Northwest, Northern, Southwest London, Wessex tools; VSTTC, Victorian state prehospital trauma triage criteria; Dutch, Dutch Field Triage Protocol; AMPT, Air Medical Prehospital Triage; HITS-NS, Head Injury Transportation Straight to Neurosurgery; Pop, population; A, Adult; P-Pediatric; GCS, Glasgow Coma Scale; Sn, sensitivity; Sp, specificity; AUC, area under the curve; CI, confidence interval; aOR, adjusted odds ratio; PPV, positive predictive value; NPV, negative predictive value

The studies included in the final synthesis were assigned a four-tier quality rating (very low, low, moderate, or high) assessed by a customized scale based off the GRADE criteria, which included the study design, number of centers, and sample size (small < 300, moderate 300–1000, or large > 1000) [13]. For example, very low quality rating was assigned to retrospective observational studies that were single center or with small sample size, and a high quality rating was reserved for interventional, controlled, multi-center studies with large sample sizes. The review protocol is available upon request from the corresponding author.

## Results

**Fig. 1:**
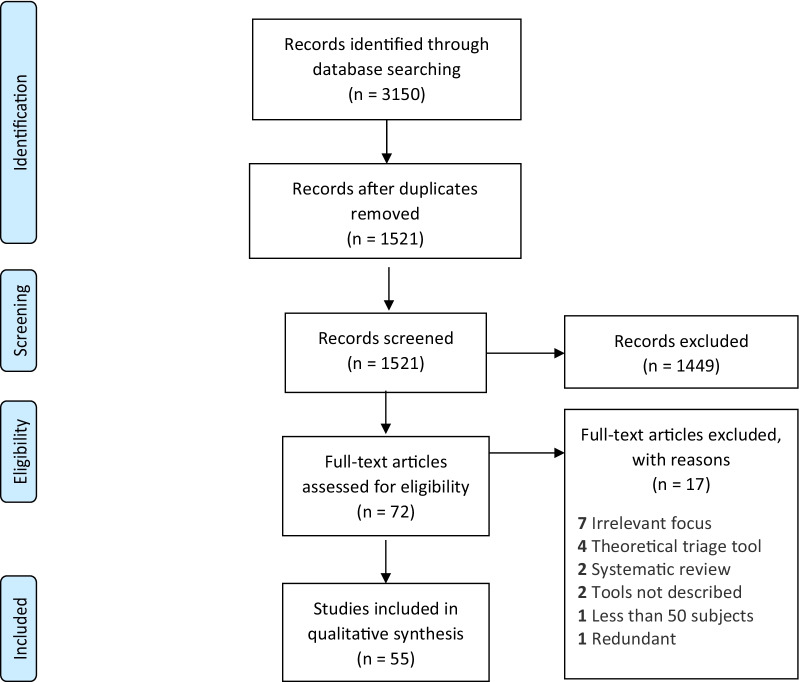
PRISMA flow diagram of articles

1521 unique articles were retrieved from database query (Fig. 1). After title and abstract screening, 72 (4.7%) met inclusion criteria, and 1449 (95.3%) were excluded. Out of 72 articles which had full-text reviews performed, 55 (3.6% of 1521 unique articles) were deemed relevant and included in the full-text qualitative synthesis. 17 articles were excluded during full-text review with reasons cited in Fig. 1. The prose format synopsis of all 55 articles can be found in Additional file 2, and a summary table of the top third highest quality studies can be found in Table 2. The summary table of all included studies can be found in Additional file 3.

### Medical conditions

Of the 55 studies included in our final analysis, 19 (35%) focused on stroke triage, 19 (35%) on trauma triage, and 15 (27%) on triage of general undifferentiated patients. Of the remaining 2 (3%) studies, one addressed infectious disease triage [14] and the other addressed triage of patients with only non-traumatic chief complaints [15].

### Location and design of studies

All studies were performed in World Bank designated high income countries, with 23 (42%) in North America, 22 (40%) in Europe, 6 (11%) in East Asia and 4 (7%) in Australia. All studies were prospective or retrospective observational cohort studies with the exception of one small randomized controlled clinical trial on stroke triage by Helwig et al. [16].

### Pediatric populations

Twelve studies within the general undifferentiated triage and trauma triage categories included the pediatric population, and four (7%) focused on pediatric patients exclusively. The Rapid Emergency Triage and Treatment System-pediatrics (RETTS-p) tool is used for general undifferentiated pediatric triage in Sweden [17, 18]. Magnusson et al. found RETTS-p sensitivity to be moderate, with a sensitivity of 66.7% and specificity of 67.0% for detecting pediatric patients with emergency care need. Two thirds of the children triaged to life threatening or potentially life threatening by RETTS-p were later identified as non-emergent by hospital providers [18]. Pediatric trauma triage studies focused on the Field Triage Decision Scheme (FTDS) for pediatric trauma in the United States [19], and several regional pediatric trauma tools employed in England [20]. Lerner et al. found that the 2011 Field Triage Guidelines for pediatric trauma had an under-triage rate of 34.8% and over-triage rate of 28.0%, concluding that the current guidelines have an unacceptably high rate of under-triage [19].

### General undifferentiated triage tools

The tools for general undifferentiated triage focused on standardized communication of level of acuity assignments between prehospital and emergency department providers. Frequently studied examples include the United Kingdom National Early Warning Score (NEWS), the Canadian Triage and Acuity Scale (CTAS) and the American Emergency Severity Index (ESI), which were originally developed for accurate triage by emergency department providers [21–23]. Tools like NEWS incorporate prehospital vital signs and level of consciousness assessments [24, 25]; other tools such as CTAS and ESI also include chief complaint, exam findings, and anticipated resource needs as part of the algorithm [26, 27]. Most studies on general triage focused on the ability of prehospital providers to use these existing tools. For example, Hoikka et al. studied the accuracy of prehospitally implemented NEWS in predicting 1-day and 30-day mortality in an unselected EMS population, and found that among 12,426 EMS calls, the high-risk NEWS group had a sensitivity of 0.80 (CI 0.74–0.86) and 0.42 (CI 0.38–0.47), respectively [24]. They concluded that prehospital NEWS may prove beneficial for assessing mortality risk within 24 h and hence immediate need for medical care, but requires further confirmatory studies. Overall, a wide variety of clinical end points were studied, including short-term, in-hospital and 30-day mortality, inter-rater reliability (e.g., between dispatch, EMS provider, and emergency room triagist), need for lifesaving interventions within hours of ED arrival, and need for intensive care unit (ICU) admission. There was significant heterogeneity of clinical end points in the articles reporting all-comer triage tools. Consequently, a single triage tool in this group with the best performance metrics could not be identified.

### Stroke triage

From 19 (35%) articles, we found 18 different stroke prehospital triage tools designed to aid with the recognition of acute stroke. The most commonly studied stroke triage tools were the Rapid Arterial Occlusion Evaluation (RACE, n = 5 studies, 26%), Cincinnati Prehospital Stroke Scale (CPSS; n = 4, 21%), Field Assessment for Stroke Triage for Emergency Destination (FAST-ED, n = 4, 21%), and Los Angeles Motor Scale (LAMS, n = 3, 16%). The authors stated the ultimate aim of prehospital stroke triage is to ensure timely transport of patients with acute ischemic stroke to designated stroke centers that have capabilities for neuroimaging, administration of thrombolytic agents and/or endovascular intervention. According to the authors, these tools also aim to channel patients presenting with stroke mimics, such as hypoglycemia or seizure, away from the major stroke centers to optimize health system resource utilization. Finally, several articles argued that stroke triage tools aim to be easy to use and efficient to administer in the prehospital setting, and to correlate well with the gold standard tools used by in-hospital providers, such as the National Institutes of Health stroke scale (NIHSS) [28]. Of the 19 articles assessed, 10 (53%) used the NIHSS as the referential standard of comparison, or as a model from which scales were derived. Clinical end points for the stroke triage studies were diverse and included: detection of large vessel occlusion (LVO), diagnosis of stroke/transient ischemic attack, tissue plasminogen activator (tPA) administration rate, stroke team activation, accurate destination triage decision, and inter-rater reliability.

The highest quality studies for stroke triage evaluated the RACE scale. RACE evaluates five items: facial palsy, upper extremity paresis, lower extremity paresis, head and gaze deviation, and aphasia/agnosia, with a total score of 0 to 9. For example, in a large prospective study in Spain, Carrera et al. validated RACE among a cohort of 1822 patients and found a sensitivity of 84% and specificity of 60% for detecting large vessel occlusion (LVO) for RACE score ≥ 5. 35% of the patients with a RACE ≥ 5 had LVO, compared with 6% LVOs with a RACE < 5 (*p* < 0.001) [29]. Jumaa et al. found that RACE ≥ 5 had a sensitivity of 77% and specificity of 75% for LVO eligible for mechanical thrombectomy among a cohort of 1147 patients in the United States [30]. Additional scales that have undergone head-to-head comparisons with RACE with comparable performance include the FAST-ED and the CPSS tools [31, 32]. The performance characteristics of FAST-ED and CPSS were assessed in 5 (26%) articles. Overall, both have comparable sensitivity (56–83%) and specificity (60–89%) for LVO prediction [31, 32].

### Trauma triage: ground EMS

A common objective of included trauma triage articles was to accurately identify injured patients that require emergent transport to designated trauma centers. The studies on trauma triage more consistently used similar end points, including trauma center need, under-triage and over-triage rates, and survival to discharge. Trauma center need was uniformly defined as Injury Severity Score (ISS) > 15, need for urgent surgical intervention, or need for intensive care unit level care.

The majority of trauma triage tools identified are based off of the Field Trauma Decision Scheme (FTDS) [33] which appears to be the de facto standard in studies originating from the USA. Since its initial publication in 1986, the FTDS has been revised five times: in 1990, 1993, 1999, 2006 and 2011. According to the articles, the FTDS uses stepwise identification of four aspects of clinical presentation involving physiologic criteria, anatomic criteria, mechanism of injury criteria, and special considerations criteria to identify patients requiring transport to a trauma center. Physiologic criteria focus on vital signs and Glasgow Coma Scale (GCS); anatomic criteria include specific severe injury patterns such as penetrating trauma, flail chest and crush injury; mechanism of injury criteria focuses on high energy mechanisms such as falls from specific height, high speed vehicular crash, and motorcycle accidents; and special considerations include extremes of age, high risk comorbidities, burns, pregnancy, and anticoagulated status [33].

The highest quality study of FTDS performance within this scoping review was conducted by Newgard et al. in 2011; it evaluated the performance characteristics of the 2006 version of FTDS, with a cohort of 122,345 injured patients evaluated and transported by EMS over a 3-year period [34]. Major trauma was defined as ISS > 15, and the overall sensitivity and specificity of the FTDS criteria for identifying major trauma patients were 86% (95% CI 85–87) and 69% (95% CI 68–69), respectively. Triage sensitivity and specificity, respectively, differed by age: 84% and 66% (0 to 17 years); 90% and 64% (18 to 54 years); and 80% and 75% (≥ 55 years). Overall, FTDS appears to have comparatively reduced sensitivity and increased specificity in detection of trauma in elderly patients.

Other frequently studied ground EMS tools included the Vittel criteria (France) [35, 36], Dutch Field Triage Protocol (Netherlands) [37, 38], and Prehospital Index (Canada) [39].

### Trauma triage: aeromedical EMS

Three studies focused on the use of trauma triage tools to decide on the utility of helicopter transport [40–42]. Brown et al. conducted a US retrospective cohort study of 258,387 trauma patients (16% transported by helicopter, remainder by ground) and found odds of increased survival to discharge for patients transported by helicopter in the following FTDS conditions: GCS < 14 (adjusted Odds Ratio 1.22); respiratory rate < 10 or > 29 (aOR 1.32), penetrating injury (aOR 1.40), or age > 55 (aoR 1.15) [41]. In 2017, Brown et al. investigated the Air Medical Prehospital Triage (AMPT) score, which awards points for low GCS, abnormal respiratory rate, unstable chest wall injury patterns, paralysis, multisystem trauma, or fulfillment of any physiologic plus anatomic criterion from FTDS. The authors found that helicopter EMS increases odds of in-hospital survival by 6.7% for patients with AMPT score ≥ 2 (Absolute Risk Reduction 1.067; 95% CI 1.040–1.083, *p* < 0.001, n = 222,827) [42].

### Trauma triage: traumatic brain injury

Two studies by Fuller et al. focused on predictive tools for triaging severe traumatic brain injury (TBI) in the field [43, 44]. The authors studied the Head Injury Transportation Straight to Neurosurgery study (HITS-NS) triage tool and London Ambulance Service major trauma triage tool and found that both had poor sensitivity (< 45%) for detection of severe TBI which was concerning for EMS providers missing TBIs [43, 44].

### Simplifying triage tools

While most trauma triage studies investigated performance characteristics of established tools, a subset attempted to identify ways to further simplify tools for EMS providers [35, 45]. These studies emphasized the challenges of designing the ideal triage tool: the design must optimize over and under-triage rates while remaining streamlined and user friendly to promote widespread adoption.

## Discussion

Our scoping review found 55 studies on prehospital triage tools published within the past decade. These tools focused on general undifferentiated, trauma, and stroke populations and all included studies originated from high-income countries. Studies predominantly sought to assess predictive accuracy of the triage tools compared to in-hospital clinical outcomes, and many studied accuracy in simplified versions of existing tools. These published triage tools are generally designed to help prehospital providers determine destination of transport, means of transport and level of acuity. These tools also appear to provide a shared language for prehospital personnel to communicate with other emergency personnel, and assist in identifying vital sign derangements and exam findings across a spectrum of age ranges to differentiate ‘acute’ and ‘non-acute’ patients.

Trauma and stroke tools comprised over two-thirds of the included articles, perhaps because of their clinical and health systems significance [46–51]. Outcomes for trauma and stroke depend on timely field recognition and are influenced by highly time sensitive interventions that are destination-dependent. Further, trauma and stroke care are regionalized in many high-income countries, therefore correct patient destination decision-making is important to study for trauma and stroke system optimization. Last, both stroke and trauma outcomes are used to drive ‘benchmarking’ for health system accreditation and funding, which may also drive their importance as a research topic.

In trauma, the US FTDS appears to be the “industry standard” triage tool used, likely reflecting that the majority of our studies were from North America, specifically, the USA [33, 52]. As the majority of tools within the trauma triage literature derive from the FTDS, this well-researched tool is a promising starting point for further simplified trauma triage tool development, such as identifying individual components that may predict clinically relevant trauma outcomes [34]. The trauma literature was relatively cohesive in that most studies used common clinical end points, which facilitates comparisons across studies.

In stroke care, while no single tool emerged as the prehospital triage ‘gold’ standard, the RACE, FAST-ED and Cincinnati Prehospital Stroke scales appear to have the highest quality data supporting their use [29–32]. The National Institutes of Health Stroke Scale was presented in multiple studies as the gold standard in-hospital tool which was used for comparison [28].

The all-comer triage literature includes a myriad of tools with varying complexity, from those that incorporate vital signs alone (e.g., NEWS), to those with complex diagnostic algorithms incorporating history and exam findings to arrive at a level of acuity designation (e.g., CTAS). No one tool emerged as a clear gold standard, and authors’ use of a wide variety of clinical end points which make cross comparisons challenging.

Research themes common to these studies include simplifying existing tools such that they are efficient and accurate for the EMS provider to derive an accurate triage decision, and to identify the most accurate tool out of a large cadre of tools currently available. Standardized reporting of clinical end points would facilitate this endeavor in future research. Additionally, we noted a paucity of articles researching implementation or assessing end user perspectives [29, 51, 53], and no studies examined costs associated with triage decisions. Qualitative studies assessing EMS provider perceptions of usefulness of prehospital triage tools, cost analyses, and implementation studies would be helpful to further our understanding of the value provided by prehospital triage tools.

Lastly, all the studies included in this scoping review were performed in a few high-income settings, and the tools may not translate well to other high-income settings or LMICs with a different healthcare configuration, infrastructure and cadres of prehospital providers [54]. Destination decision making would need to be locally-determined, especially in LMICs where specialty diagnostic (e.g., computed tomography scanners) and therapeutic resources (e.g., tPA) may be even more scarce. Further, triage tools may need to be tailored based upon regional injury and illness patterns. For example, prehospital triage of obstetric emergencies was notably missing from our review. Jenson et al. performed a systematic review of emergency department (i.e. in hospital) triage tools in LMICs and identified the South Africa Triage Scale (SATS), modified Early Warning Score and the Australasian Triage Scale as promising tools that had been validated across multiple studies in LMIC settings [8]. SATS has been implemented in the prehospital setting in South Africa and studies analyzing real-world performance characteristics, while on-going, are yet to be published [55]. In 2021, Mould-Millman et al. published a theoretical assessment-based validation study of SATS among EMS providers in South Africa. Among 102 EMS providers who performed triage using clinical vignettes, the final SATS triage color was accurately determined in 56.5%, under-triaged in 29.5% and over-triaged in 13.1%, demonstrating good inter-rater reliability but poor validity [56].

In recent years, prehospital care has received increased recognition in international health policy. Data extrapolated from the Global Burden of Disease study show that 24 million lives are lost each year in LMICs due to conditions sensitive to prehospital and emergency care. Ischemic heart disease, cerebrovascular accidents, and unintentional injuries are the largest contributors to morbidity and mortality in these settings [6, 57]. In 2019, delegates to the 72nd World Health Assembly adopted a resolution to strengthen emergency and trauma care systems and prehospital care was highlighted as an essential component [58]. Prehospital triage tools are a key building block for quality and safety assurance in the development of novel EMS systems [59]. It is our hope that this scoping review has provided a valuable framework for what is known thus far, and that further research will be done to advance the field.

The authors acknowledge the following limitations of this scoping review. First, the review was limited to English language publications. This may have excluded triage tools published in non-English journals. The review was limited to only peer-reviewed published literature; it is likely that white papers and other non-peer-reviewed papers discuss additional triage tools currently in use. The review protocol was not pre-registered but otherwise followed the PRISMA-ScR recommendations [60]. We included articles with sample sizes or 50 or more cases, which was arbitrary, but intended to select for larger sample size articles from which more compelling conclusions could potentially be drawn. Lastly, inherent to this study’s design as a scoping review, the authors were unable to draw quantitative conclusions about the performance characteristics of the tools presented.

## Conclusions

This scoping review found that the majority of literature on prehospital triage focused on trauma and stroke specifically, with a few reports on triage tools for general undifferentiated patients. Much of this body of work originates from high income countries. The Field Triage Decision Scheme for trauma, and the Rapid Arterial Occlusion Evaluation for stroke, are especially well studied tools which may serve as tools for emerging EMS systems or as good starting points for simplified adaptations for established EMS systems. We found no single universally accepted ‘standard’ prehospital triage tool. Future research should focus on implementation analysis and real-world application of these tools. Additionally, research efforts should focus on the development of a single universal triage tool that can be adapted for a variety of contexts.

## Supplementary Information

**Additional file 1.** Database search strategies.

**Additional file 2.** Prose summary of included articles.

**Additional file 3.** Summary table of included articles.

## Data Availability

All data generated or analyzed during this study are included in this published article [and its supplementary information files].

## Abbreviations

AMPT: Air Medical Prehospital Triage
aOR: Adjusted odds ratio
CPSS: Cincinnati Prehospital Stroke Scale
CTAS: Canadian Triage and Acuity Scale
EKG: Electrocardiogram
EMS: Emergency medical services
ESI: Emergency Severity Index
FAST-ED: Field Assessment for Stroke Triage for Emergency Destination
FTDS: Field Triage Decision Scheme
GCS: Glasgow Coma Scale
HITS-NS: Head Injury Transportation Straight to Neurosurgery
ICU: Intensive care unit
LAMS: Los Angeles Motor Scale
LMICs: Low and middle income countries
LVO: Large vessel occlusion
NEWS: National Early Warning Score
NIHSS: National Institutes of Health Stroke Scale
RACE: Rapid Arterial Occlusion Evaluation
RETTS-p: Rapid Emergency Triage and Treatment System-pediatrics
SATS: South Africa Triage Scale
STEMI: ST elevation myocardial infarction
TBI: Traumatic brain injury
tPA: Tissue plasminogen activator
