# Supplementary material for: Prehospital triage tools across the world: a scoping review of the published literature
Source: Scand J Trauma Resusc Emerg Med. 2022 Apr 27;30:32. doi: 10.1186/s13049-022-01019-z (PMC9044621; doi:10.1186/s13049-022-01019-z)
Supplement: Supplementary file 1 — Additional file 1. Database search strategies. [file 13049_2022_1019_MOESM1_ESM.docx]

| ***Additional File 1: Database Search Strategies*** | | |  |
| --- | --- | --- | --- |
| **MEDLINE (via Ovid MEDLINE® and Epub Ahead of Print, In-Proce & Other Non-Indexed Citation, Daily and Verion®, 1946 to present)** | | |  |
| Search date = 12/23/2019 | | |  |
| 1 | ambulance*.ab,ti. | |  |
| 2 | emergency medical technician*.ab,ti. | |  |
| 3 | EMT*.ab,ti. | |  |
| 4 | paramedic*.ab,ti. | |  |
| 5 | prehospital*.ab,ti. | |  |
| 6 | pre-hospital*.ab,ti. | |  |
| 7 | or/1-6 | |  |
| 8 | exp "Triage"/ | |  |
| 9 | triag*.ab,ti. | |  |
| 10 | or/8-9 | |  |
| 11 | 7 and 10 | |  |
| 12 | 11 and English.la. | |  |
| 13 | limit 12 to yr="2009 -Current" | |  |
| 14 | 13 not (exp case reports/ or exp "review"/ or exp meta-analysis/ or exp "systematic review"/ or exp comment/ or exp editorial/ or exp letter/) | |  |
| 15 | remove duplicates from 14 | |  |
| **Embase (via Elsevier, Embase.com, 1947 to present)** | | |  |
| Search date = 12/23/2019 | | |  |
| 1 | ambulance*:ab,ti | |  |
| 2 | 'emergency medical technician*':ab,ti | |  |
| 3 | emt*:ab,ti | |  |
| 4 | paramedic*:ab,ti | |  |
| 5 | prehospital*:ab,ti | |  |
| 6 | 'pre hospital*':ab,ti | |  |
| 7 | #1 OR #2 OR #3 OR #4 OR #5 OR #6 | |  |
| 8 | triag*:ab,ti | |  |
| 9 | #7 AND #8 | |  |
| 10 | #9 AND [english]/lim | |  |
| 11 | #10 AND [2009-2020]/py | |  |
| 12 | #11 NOT ('case report'/exp OR 'case study'/exp OR 'review'/exp OR 'letter'/exp OR 'editorial'/exp OR 'note'/exp) | |  |
| 13 | #12 AND ('article'/it OR 'article in press'/it OR 'review'/it) | |  |
| **Web of Science Core Collection (via Clarivate Analytics, including Science Citation Index Expanded and Social Sciences Citation Index, 1974 to present)** | | |  |
| Search date = 12/23/2019 | | |  |
| 1 | TS="ambulance*" | |  |
| 2 | TS="emergency medical technician*" | |  |
| 3 | TS="EMT*" | |  |
| 4 | TS="paramedic*" | |  |
| 5 | TS="prehospital*" | |  |
| 6 | TS="pre-hospital*" | |  |
| 7 | #1 OR #2 OR #3 OR #4 OR #5 OR #6 | |  |
| 8 | TS="triag*" | |  |
| 9 | #7 AND #8 | |  |
| 10 | (#9) AND LANGUAGE: (English) | |  |
| 11 | (#10) AND LANGUAGE: (English); Timespan=2009-2019 | |  |
| 12 | (#11) AND LANGUAGE: (English) AND DOCUMENT TYPES: (Article OR Correction OR Review); Timespan=2009-2019 | |  |
| **Summary of database search strategies** | | | |
| **Database** | | **# Results** | |
| Ovid MEDLINE | | 1077 | |
| Embase | | 902 | |
| Web of Science | | 1171 | |
| Subtotal | | 3150 | |
| Duplicates removed | | 1629 | |
| **Total unique results** | | **1521** | |
