# Supplementary material for: Prehospital triage tools across the world: a scoping review of the published literature
Source: Scand J Trauma Resusc Emerg Med. 2022 Apr 27;30:32. doi: 10.1186/s13049-022-01019-z (PMC9044621; doi:10.1186/s13049-022-01019-z)
Supplement: Supplementary file 2 — Additional file 2. Prose summary of included articles. [file 13049_2022_1019_MOESM2_ESM.docx]

***Additional File 2: Prose Summary of Included Articles***

**Anadani M, Almallouhi E, Wahlquist AE, Debenham E, Holmstedt CA. The Accuracy of Large Vessel Occlusion Recognition Scales in Telestroke Setting. Telemedicine Journal and e-Health. 2019;25(11):1071-6.**

This study aimed to assess the accuracy of the most commonly used large vessel occlusion (LVO) recognition scales in the prehospital setting. The National Institutes of Health Stroke Scale (NIHSS) was calculated and documented by a consulting stroke neurologist during telestroke consults and used to calculate the following scales: 3-item stroke scale (3I-SS), Rapid Arterial Occlusion Evaluation Scale (RACE), Cincinnati Prehospital Stroke Severity Scale (CPSSS), Prehospital Acute Stroke Severity Scale (PASS), and the Field Assessment Stroke Triage for Emergency Destination (FAST-ED) stroke scale.

A retrospective chart review was performed of all consecutive telestroke patients who were transferred to the rural Medical University of South Carolina hub for suspected LVO during the period from May 2014 to March 2018. Using published cutoffs for each scale, the sensitivity, specificity, accuracy, positive and negative predictive values, false positive rate (FPR), and false negative rate (FNR) of each score were calculated. Secondarily, all possible cutoff values were considered for a given scale to identify the cutoff that maximized the sum of sensitivity and specificity.

A total of 439 patients were included in the final analysis. A total of 48.5% of patients had an LVO confirmed on computed tomography angiogram. RACE score (using cutoff of greater than or equal to 5) had the highest accuracy for predicting LVO (78%), with a FNR of 11% and FPR of 33%. All five derived prehospital LVO recognition scores had at least 10% FNR. In contrast, the NIHSS (cutoff of greater than or equal to 6) had a 3% FNR and 73% FPR (implying unnecessary transfer). Better diagnostic tools that maximize sensitivity with acceptable specificity are urgently needed.

**Andsberg G, Esbjornsson M, Olofsson A, Lindgren A, Norrving B, von Euler M. PreHospital Ambulance Stroke Test - pilot study of a novel stroke test. Scandinavian Journal of Trauma, Resuscitation and Emergency Medicine. 2017;25(1):37.**

The objective of this study was to evaluate the performance characteristics of PreHospital Ambulance Stroke Test (PreHAST) in terms of sensitivity for stroke/transient ischemic attack (TIA) detection in the prehospital setting, along with ability to communicate stroke severity similar to the National Institutes of Health Stroke Scale (NIHSS).

The PreHAST is an eight-item test based on NIHSS which scores stroke severity from 0–19 points. It involves evaluation of neurological signs with high prevalence in stroke as compared to stroke mimics in the prehospital screening (e.g., Level of consciousness, gaze deviation, facial palsy, arm/leg paresis, dysarthria, hemianopia and sensory loss).

Ambulance nurses in rural Sweden prospectively applied PreHAST to assess adult patients with suspected stroke in the prehospital setting. Of 69 patients included in the study, 26 had stroke/TIA and 43 had other diagnoses. There was a sensitivity of 100% (95% CI 87-100%) and a specificity of 40% (95% confidence interval (CI) 25-56%) for Stroke/TIA when a positive score in any PreHAST item (PreHAST score 1–19 points) was found after prehospital assessment in patients with suspected stroke. Furthermore, the positive and negative predictive value was 50% and 100%, respectively, for a positive PreHAST score. The receiver operating characteristic (ROC) analysis showed an area under the curve (AUC) of 0.77 (95%CI; 0.66-0.88). In conclusion, PreHAST is a new screening test of stroke adapted for ambulance services that in addition to high sensitivity for stroke, provides a grading system with increasing specificity with higher scores.

**Ardolino A, Cheung CR, Lawrence T, Bouamra O, Lecky F, Berry K, et al. The accuracy of existing prehospital triage tools for injured children in England: an analysis using emergency department data. Emergency Medicine Journal. 2015;32(5):397-400.**

The objective of this study is to build on previous work using trauma registry data based in the Trauma Audit and Research Network (TARN) to assess the performance characteristics of pediatric prehospital trauma triage tools currently in use or under development in England. This study compares 8 tools: East Midlands standard operating procedure, London triage tool, North West, Northern, South West London and Surrey, Pediatric triage score, Pediatric triage tape, and the Wessex triage tool.

Retrospective clinical data of 2934 patient records from four emergency departments in England were used to interrogate each of the triage tools. Inclusion criteria were people aged below 16 years sustaining injury or trauma between January 2011 and June 2012 who attended the emergency department without using an ambulance. The constituent discriminators for each tool were then retrospectively applied to the dataset of injured children from each emergency department to derive sensitivity, specificity, positive predictive value, negative predictive value and likelihood ratio measures for each tool’s efficacy in identifying seriously injured children (defined as those with ISS >15).

Three of the tools fulfilled acceptable criteria for both over and under-triage (East Midlands, North West and Northern), with over-triage rates of 18%, 21% and 19%, respectively, and under-triage rates of 0% in each. The other five tools fell below the target sensitivity of 95%. All eight tools had acceptable specificity (with results between 79% and 99%). In this population of children with minor to moderate injuries, the prehospital triage tools in common use or in development across England all show high sensitivity and moderate specificity. Five of the eight tools analyzed (London, South West London/Surrey, Wessex, Pediatric trauma score and Pediatric triage tape) show a tendency to under-triage.

**Barnett AS, Wang NE, Sahni R, Hsia RY, Haukoos JS, Barton ED, et al. Variation in prehospital use and uptake of the national Field Triage Decision Scheme. Prehospital Emergency Care. 2013;17(2):135-48.**

The American College of Surgeons Committee on Trauma and the Centers for Disease Control’s National Trauma Triage Protocol: Field Triage Decision Scheme (FTDS) is a national guideline that has been implemented widely for prehospital emergency medical services (EMS) and trauma systems. The objective of this study is to describe the use of field triage criteria by EMS personnel in six regions of the Western United States, including the timing of guideline uptake and the use of non-guideline criteria.

This study used a retrospective cohort of injured children and adults transported by 48 EMS agencies to 105 hospitals in six Western U.S. regions from 2006 through 2008. Descriptive statistics were used to compare the frequency of triage criteria use and to evaluate the timing of guideline uptake across multiple versions of the guidelines. A total of 260,027 injured patients were evaluated and transported by EMS over the three-year study period, of whom 46,414 (18%) met at least one field triage criterion and formed the primary sample for analysis. The three most common criteria cited were EMS provider judgment (26%), age <5 or >55 years (10%), and Glasgow Coma Scale (GCS) score <14 (9%). Of the 33 criteria in use, 5 (15%) were previously retired from the guidelines and 7 (21%) were never included in the guidelines. 11,048 (24%) of patients had more than 1 criteria applied (range 1 – 21).

This study found large variation between regions in the frequency and type of field triage criteria used. Field uptake of guideline revisions appears to be slow and variable, suggesting opportunities for improvement in dissemination and implementation of updated guidelines.

**Bergs J, Sabbe M, Moons P. Prehospital stroke scales in a Belgian prehospital setting: a pilot study. European Journal of Emergency Medicine. 2010;17(1):2-6.**

The aim of this study was to directly compare the diagnostic value of the Cincinnati Prehospital Stroke Scale (CPSS), Face Arm Speech Test (FAST), Los Angeles Prehospital Stroke Screen (LAPSS), and the Melbourne Ambulance Stroke Screen (MASS) for identifying patients with a stroke in a specific Belgian emergency medical service (EMS).

The CPSS and FAST both evaluate the presence or absence of facial palsy, unilateral arm weakness, and speech impairment. If at least one item is present, stroke is suspected. The LAPSS and the MASS incorporate history criteria and a blood glucose measurement in addition to clinical assessment. The MASS contains the same items as the LAPSS with the addition of a speech impairment evaluation.

This prospective study was conducted from December 2005 through April 2006 in the emergency department (ED) of the University Hospitals in Leuven, Belgium. A questionnaire for every transported patient was used which contained 10 questions: four history questions, a blood glucose measurement, and a limited clinical examination. The results for each scale were compared with the diagnosis at discharge to determine prevalence, sensitivity, specificity, positive predictive value, negative predictive value, likelihood ratio, and accuracy.

Seventy patients were transported to the ED of the University Hospitals Leuven. The EMS staff completed a questionnaire for 31 (44%) patients. Finally, 19 (61.3%) patients had a diagnosis of a stroke. The FAST and CPSS demonstrate a high sensitivity (95%) but a lower specificity (33%). The sensitivity of the LAPSS and MASS was lower (74%), but the specificity increased (83 and 67%). Items investigating unilateral facial paralysis and unilateral loss/absence of motor response in upper extremities seemed to be most discriminating between the stroke group (68–78%) and the non-stroke group (17%), suggesting that items related to clinical assessment are more important in stroke recognition than history items. Further research is needed to determine the most adequate stroke scale

**Brown JB, Forsythe RM, Stassen NA, Gestring ML. The National Trauma Triage Protocol: can this tool predict which patients with trauma will benefit from helicopter transport? The Journal of Trauma and Acute Care Surgery. 2012;73(2):319-25.**

The American College of Surgeons Committee on Trauma and the Centers for Disease Control jointly developed the National Trauma Triage Protocol: Field Triage Decision Scheme (FTDS) to identify trauma patients who would benefit from trauma center care. The FTDS is based on the stepwise identification of four aspects of clinical presentation involving physiologic criteria (PHY), anatomic criteria (ANA), mechanism of injury criteria, and special considerations criteria that are evaluated in a sequential fashion to identify patients who should be transported to a trauma center. Physiologic criteria focus on vital signs and Glasgow Coma Scale (GCS); anatomic criteria include specific injury patterns such as penetrating trauma, flail chest, crush injury, etc; mechanism of injury criteria focuses on high risk patterns such as falls from specific height, high speed vehicular crash, motorcycle accident etc; and special considerations include extremes of age, comorbidities, burns, pregnancy, anticoagulated status, etc.

The study objective was to determine if the criteria included in the FTDS could be used by field emergency medical services providers to predict which patients would benefit from helicopter transport after injury. Subjects in the United States transported by helicopter or ground transport from the scene of injury in 2007 were identified using the National Trauma Databank version 8. Criteria from the stepwise FTDS available in the data set were collected. Subgroups of patients who met specific triage criteria were evaluated using logistic regression to determine if transport modality was an independent predictor of survival after controlling for demographics, injury severity, prehospital time, and presence of other FTDS triage criteria. Standard test characteristics were calculated for each criterion to predict trauma center need (TCN). The performance of triage criteria to predict TCN was compared between the groups using independent receiver operating characteristic area under the curve analysis.

There were 258,387 subjects transported either by helicopter (16%) or by ground (84%). Helicopter transport was an independent predictor of survival to discharge when compared with ground transport in the presence of penetrating injury, GCS score of less than 14, respiratory rate of less than 10 breaths per minute or more than 29 breaths per minute, and age of more than 55 years. In addition, subjects that had any one PHY plus any one ANA had a survival advantage if transported by helicopter when compared with ground transport. In conclusion, patients who meet certain triage criteria in the field seem to have an independent survival benefit if transported to a trauma center by helicopter. Furthermore, these criteria are highly specific and more reliably predict TCN in the helicopter transport group.

**Brown JB, Gestring ML, Guyette FX, Rosengart MR, Stassen NA, Forsythe RM, et al. External validation of the Air Medical Prehospital Triage score for identifying trauma patients likely to benefit from scene helicopter transport. The Journal of Trauma and Acute Care Surgery. 2017;82(2):270-9.**

The Air Medical Prehospital Triage (AMPT) score was developed to identify injured patients who may benefit from scene helicopter emergency medical services (HEMS) transport. One point is assigned for Glasgow Coma Scale<14, respiratory rate <10 or >29, unstable chest wall fracture, suspected hemothorax or pneumothorax, paralysis, or multisystem trauma involving 3 or more anatomic regions; 2 points are assigned if patient has any 1 physiologic criterion plus any 1 anatomic criterion present from American College of Surgeons Committee on Trauma national field triage guidelines. If the summation of points is two or greater, the patient should be considered for helicopter transport.

The study objective was to validate the effectiveness of the AMPT score in identifying patients with a survival benefit from HEMS. Patients 16 years or older undergoing scene HEMS or ground EMS (GEMS) transport in the Pennsylvania Trauma Outcomes Study registry from 2000 to 2013 were included. Patients with 2 or higher AMPT score points were triaged to HEMS, while those with less than 2 points were triaged to GEMS. Multilevel Poisson regression determined the association of survival with actual transport mode across AMPT score triage assignments, adjusting for demographics, mechanism, vital signs, interventions, and injury severity. There were 222,827 patients included. For patients triaged to GEMS by the AMPT score, actual transport mode was not associated with survival (adjusted relative risk, 1.004; 95% confidence interval, 0.999–1.009; p = 0.08). For patients triaged to HEMS by the AMPT score, actual HEMS transport was associated with a 6.7% increase in the relative probability of survival (adjusted relative risk 1.067; 95% confidence interval, 1.040–1.083, p < 0.001). This study is the first to externally validate the AMPT score, demonstrating the ability of this tool to reliably identify trauma patients most likely to benefit from HEMS transport.

**Brown JB, Stassen NA, Bankey PE, Sangosanya AT, Cheng JD, Gestring ML. Mechanism of Injury and Special Consideration Criteria Still Matter: An Evaluation of the National Trauma Triage Protocol. Journal of Trauma-Injury Infection and Critical Care. 2011;70(1):38-45.**

The American College of Surgeons Committee on Trauma and the Centers for Disease Control jointly developed the National Trauma Triage Protocol: Field Triage Decision Scheme (FTDS), to identify trauma patients who would benefit from trauma center care. The FTDS is based on the stepwise identification of four aspects of clinical presentation including physiologic criteria (PHY), anatomic criteria (ANA), mechanism of injury criteria, and special considerations criteria that are evaluated in a sequential fashion to identify patients who should be transported to a trauma center. Physiologic criteria focus on vital signs and Glasgow Coma Scale; anatomic criteria include specific injury patterns such as penetrating trauma, flail chest, crush injury, etc; mechanism of injury criteria focuses on high risk patterns such as falls from specific height, high speed vehicular crash, motorcycle accident etc; and special considerations include extremes of age, comorbidities, burns, pregnancy, anticoagulated status, etc.

The study objective was to analyze whether trauma center need was accurately predicted solely by the physiologic (PHY) and anatomic (ANA) criteria of the CDC National Trauma Triage Protocol. Trauma patients aged 18 years and older were identified in the National Trauma Data Bank (2002–2006). Trauma Center Need (TCN) was defined as Injury Severity Score (ISS) >= 15, intensive care unit admission, or need for urgent surgery. Logistic regression was performed to determine independent association of criteria with outcomes. A total of 1,086,764 subjects were identified. PHY criteria as a whole were more sensitive (32% vs. 26%) and specific (91% vs. 86%) than ANA criteria with lower rates of overtriage and undertriage. When the PHY and ANA criteria were applied sequentially as designed in the triage protocol, sensitivity rose to 49% sensitivity and specificity fell to 78% for identifying TCN. Undertriage using the first two steps of the triage protocol was 51%. Undertriage for TCN based on actual treating trauma center level was 11%. Current PHY and ANA criteria are highly specific for TCN but result in a high degree of undertriage when applied independently. This implies that additional factors such as mechanism of injury and the special considerations included in the Centers for Disease Control decision algorithm contribute significantly to the effectiveness of this field triage tool.

**Buschhorn HM, Strout TD, Sholl JM, Baumann MR. Emergency medical services triage using the emergency severity index: is it reliable and valid? Journal of Emergency Nursing. 2013;39(5):e55-63.**

The Emergency Severity Index is a 5-tiered system that uses both illness severity and anticipated resource needs of the patient to stratify patients into triage categories. Level 1 is assigned for patients requiring immediate life-saving intervention; level 2 is for those in high risk situations, and those having altered mental status, severe pain/distress, or with danger zone vitals (using specific cut offs for heart rate, respiratory rate and oxygen saturation by age); level 3 is for those with many anticipated resource needs; level 4 is those requiring one anticipated resource; level 5 is those requiring no resources. Resources include laboratory analysis, imaging, intravenous or intramuscular medications, specialty consultations, and/or bedside procedures such as laceration repair or conscious sedation. History and physical, point-of-care testing, oral medications, tetanus vaccine updates, and simple procedures like dressing changes and provision of crutches/slings are not classified as resources in the ESI algorithm.

This prospective, single center observational study evaluated interrater reliability in ESI scores assigned by prehospital personnel and emergency department nurses (RNs). Interrater reliability, differences based on provider experience, and validity of EMS triage assignments (sensitivity and specificity) were evaluated. Seventy-five paired, blinded triages were completed. Overall concordance between EMS providers and RNs was 0.41 (95% confidence interval [CI], 0.26-0.56). Concordance for EMS providers with less experience was 0.52 (95% CI, 0.26-0.78), and for those with more experience was 0.35 (95% CI, 0.16-0.54). Sensitivity ranged from 0% to 68% and specificity ranged from 68% to 97%. There was moderate concordance between EMS and RN ESI triage assignments. EMS sensitivity for correct acuity assignment was generally poor, whereas specificity for correctly not assigning a particular level was better.

**Carr K, Yang Y, Roach A, Shivashankar R, Pasquale D, Serulle Y. Mechanical Revascularization in the Era of the Field Assessment Stroke Triage for Emergency Destination (FAST-ED): A Retrospective Cohort Assessment in a Community Stroke Practice. Journal of Stroke and Cerebrovascular Diseases. 2019:104472.**

The field assessment for stroke triage (FAST-ED) prehospital triage tool, is one of many stroke severity scales designed to decrease time to diagnosis in the field and optimize patient triage to comprehensive stroke centers. The FAST-ED scale (*F*acial Palsy [scored 0-1], *A*rm weakness [0-2], Speech changes [0-2], *T*ime [documentation for decision making but no points], *E*ye deviation [0-2], and *D*enial/neglect [0-2]) was designed based on items of the National Institutes of Health Stroke Scale (NIHSS) with higher predictive value for large vessel occlusion stroke.

The study objective was to assess the impact of the implementation of the FAST-ED triage tool on the activation of the stroke intervention team in a community stroke treatment practice in Florida, United States. This was a retrospective chart review of admitted stroke alert patients brought in to a single center by EMS between March 2017 and September 2018. The association between EMS-documented FAST-ED scores and impact on time to revascularization as well as the association between FAST-ED scores and the presence of emergent large vessel occlusion were analyzed. Admission FAST-ED scores for the overall cohort of 402 patients was 3.3 ± 2.3 (mean ± SD) versus 5.1 ± 2.1 (mean ± SD) in patients with radiographically identified emergent large vessel occlusion. In general, there was a direct but weak correlation between FAST-ED scores and National Institutes of Health Stroke Scale severity (Spearman rank test p = 0.45, p<0.001) on admission. Receiver operator curve analysis for the patient population suggested a FAST-ED cut off greater than or equal to 4 (Sensitivity 80%; Specificity 68%; PPV 40 %), and NIHSS greater than or equal to 12 (Sensitivity 76%; Specificity 74%; PPV 41%) for predicting an emergent vessel occlusion.

There was a statistically significant improvement in interventional team activation times in favor of the FAST-ED cohort (74 minutes vs. 110 minutes, p < 0.05). FAST-ED implementation demonstrated a statistically significant improvement on stroke team activation times for patients who are candidates for mechanical revascularization.

**Carrera D, Gorchs M, Querol M, Abilleira S, Ribo M, Millan M, et al. Revalidation of the RACE scale after its regional implementation in Catalonia: a triage tool for large vessel occlusion. Journal of Neurointerventional Surgery. 2019;11(8):751-6.**

The RACE scale is a prehospital tool that aims to detect acute stroke patients with a high probability of having a large vessel occlusion (LVO). The RACE scale evaluates five items: facial palsy, upper extremity paresis, lower extremity paresis, head and gaze deviation, and aphasia/agnosia, with a total score of 0–9.

The objective of this study was to revalidate the RACE scale after its region-wide implementation in Catalonia, Spain, and to analyze geographical differences in access to endovascular treatment (EVT). Prospective data from the Stroke Code Catalan registry was collected for all stroke code activations. The RACE score evaluated by emergency medical services, time metrics, final diagnosis, presence of LVO, and type of revascularization treatment were registered. Sensitivity, specificity, and area under the curve (AUC) for the RACE cut-off value ≥5 for identification of both LVO and eligibility for EVT were calculated. The RACE scale was evaluated in the field in 1822 patients, showing a strong correlation with the subsequent in-hospital evaluation of the National Institute of Health Stroke Scale (r=0.74, P<0.001). A RACE score ≥5 detected LVO with a sensitivity 0.84 and specificity 0.60 (AUC 0.77). Patients with RACE ≥5 harbored a LVO and received EVT more frequently than RACE <5 patients (LVO 35% vs 6%; EVT 20% vs 6%; all P<0.001). This large validation study confirms RACE accuracy to identify stroke patients eligible for EVT.

**Cassignol A, Markarian T, Cotte J, Marmin J, Nguyen C, Cardinale M, et al. Evaluation and Comparison of Different Prehospital Triage Scores of Trauma Patients on In-Hospital Mortality. Prehospital Emergency Care. 2019a;23(4):543-50.**

Several prehospital major trauma patient triage scores have been developed, including the triage revised trauma score (T-RTS), Vittel criteria, Mechanism/Glasgow Coma Scale/Age/Systolic blood pressure score (MGAP), and the new trauma score (NTS). These scoring schemes allow a rapid and accurate prognostic assessment of the severity of trauma. The scoring systems are used as follows. T-RTS is scored based on the Glasgow Coma Scale, systolic blood pressure, and respiratory rate. T-RTS ranges from 0 (no signs of life) to 12 (normal vital functions). The Vittel criteria include 5 parameters: abnormal vital signs including abnormal GCS, high risk mechanism of injury, certain anatomical injuries, high degree of prehospital resuscitation required, and special considerations (age > 65, pregnancy, comorbidities). The presence of only one Vittel criterion justifies patient care in a Level 1 trauma center. The MGAP score uses age, Glasgow Coma Scale, systolic blood pressure, and type of trauma (blunt or penetrating trauma), to group patients into low (23-29), intermediate (18-22) or high risk for mortality (3-17). The NTS combines oxygen saturation, systolic blood pressure and the Glasgow Coma Scale to predict the risk of mortality as follows: very high risk (3–5), high risk (6–11), intermediate risk (12–17), and low risk (18–23).

The study objective was to compare these four scores with 30 day in-hospital mortality predictions in a cohort of consecutive admitted trauma patients. This was a single center retrospective study performed over a 4 year period (2013-2016) in a Level 1 trauma center in southern France. The diagnostic performance of each score to predict in-hospital mortality was assessed using receiver operating characteristic analysis. A total of 1,001 patients were included in the analysis, 238 (24%) females, aged 43 ± 19 years with ISS 15 ± 13. The area under the curve for each score was as follows: T-RTS, AUC= 0.84, [0.82–0.87]; Vittel criteria, AUC = 0.87 [0.85–0.89]; MGAP score, AUC = 0.91 [0.89–0.92] and NTS, AUC = 0.90 [0.88–0.92]. To test the ability of each score to be used for triage, sensitivity, specificity, positive predictive value, and negative predictive value were calculated at their usual thresholds (T-RTS <12, Vittel criteria >= 1, MGAP < 23, NTS < 18). With the current thresholds, the sensitivity, specificity, positive and negative predictive values of these scores were 91%, 35%, 10%, 98% for T-RTS, 100%, 2%, 8%, 100% for Vittel criteria, 91%, 71%, 24%, 99% for MGAP score, and 82%, 86%, 33%, 98% for NTS. Only Vittel's criteria achieved undertriage rates below 5% as recommended by the American College of Surgeons Committee on Trauma (ACS-COT). The MGAP and NTS scores had better performance compared with the T-RTS. Including the calculation of MGAP or NTS scores with the Vittel criteria would reduce the risk of overtriage in the Level 1 trauma centers by further directing patients at low risk of death to a lower-level trauma facility.

**Cassignol A, Marmin J, Cotte J, Cardinale M, Bordes J, Pauly V, et al. Correlation between field triage criteria and the injury severity score of trauma patients in a French inclusive regional trauma system. Scandinavian Journal of Trauma, Resuscitation and Emergency Medicine. 2019b;27(1):71.**

In France, the pre-hospital field triage of trauma patients is currently based on the Vittel criteria algorithm which was originally created in 2002 before the stratification of trauma centers. The Vittel criteria include 5 parameters: abnormal vital signs including abnormal GCS, high risk mechanism of injury (also called kinetic elements), certain anatomical injuries, high degree of prehospital resuscitation required, and special considerations (age > 65, pregnancy, comorbidities). The presence of only one Vittel criterion justifies patient care in a Level 1 trauma center.

The study objective was to evaluate the correlation between each Vittel field triage criterion and the presence of an Injury Severity Score of greater than 15, mortality within 30 days, and admission to the intensive care unit. This was a single center prospective observational study in France. Of the 1373 patients in the registry, 1151 were included in the analysis with a mean age of 43 years (± 19) and a median ISS of 13 (IQR = 5–22), where 887 (77%) were male. Nine of the 24 Vittel criteria were associated with an ISS > 15. For prediction of 30 day mortality, kinetic elements had a sensitivity of 77%, specificity of 13%, positive predictive value of 7% and negative predictive value of 86%. In a multivariate analysis, no criterion related to kinetic elements was significantly correlated with an ISS > 15, mortality within 30 days, or admission to intensive care. Criteria related to physiological variables, pre-hospital resuscitation, and physical injuries were the most relevant to predicting the severity of a trauma patient’s condition, while kinetic variables were not.

**Chenkin J, Gladstone DJ, Verbeek PR, Lindsay P, Fang J, Black SE, et al. Predictive value of the Ontario prehospital stroke screening tool for the identification of patients with acute stroke. Prehospital Emergency Care. 2009;13(2):153-9.**

The Ontario Prehospital Stroke Screening Tool consists of three inclusion criteria (unilateral weakness, slurred speech or muteness, and facial droop), a two-hour time limit from symptom onset, and six exclusion criteria. The tool’s exclusion criteria were designed to exclude patients with stroke mimics (hypoglycemia, seizure), patients needing emergent intervention (Canadian Triage and Acuity Scale Level 1, Glasgow Coma Scale score less than 10), and patients ineligible for fibrinolysis (symptoms resolved, terminally ill or palliative).

The study objective was to determine the positive predictive value (PPV) of the Ontario Prehospital Stroke Screening Tool for identification of acute stroke at a single stroke center. This was a retrospective analysis of consecutive patients transported to a regional stroke center under the prehospital acute stroke protocol over a 12-month period. Final diagnoses, treatments, and outcomes were abstracted from a provincial registry. Rates of fibrinolysis were compared with those for the 12-month period prior to implementation of the stroke protocol. 325 patients were triaged under the emergency medical services (EMS) acute stroke protocol over the study period. The PPV was 89.5% (95% confidence interval [CI]: 85.7–92.7%) for acute stroke. The rate of administration of tissue plasminogen activator (tPA) for all patients with suspected stroke increased from 5.9% to 10.1% (p = 0.04). The Ontario Prehospital Stroke Screening Tool had a high PPV for acute stroke. Following implementation of a citywide acute stroke protocol using this screening tool, there was an increase in the number of patients who were eligible for and received fibrinolysis at the stroke center.

**Cox S, Currell A, Harriss L, Barger B, Cameron P, Smith K. Evaluation of the Victorian state adult pre-hospital trauma triage criteria. Injury. 2012;43(5):573-81.**

The Victorian adult pre-hospital trauma triage criteria is modeled off the Centers for Disease Control National Trauma Triage Protocol: Field Triage Decision Scheme and consists of physiological, anatomical and mechanistic criteria to determine need for transport to a level I trauma center.

The study objective was to evaluate the performance of the Victorian prehospital trauma triage criteria in discriminating between confirmed major trauma patients and non-major trauma patients and to propose refined criteria to improve under and over-triage rates. Major trauma was defined at hospital discharge as one or more of: death, Injury Severity Score > 15, ICU ventilation or urgent surgery.

The study was conducted in Melbourne, Victoria and consisted of patients age 16 and older. The data was sourced from the pre-hospital Victorian Ambulance Clinical Information System and the Victorian State Trauma Registry from October 2006 through September 2007. The triage criteria were evaluated using multivariate logistic regression and classification tree modeling. Diagnostic statistics, including sensitivity and specificity were calculated to assess triage performance. Of 45,332 trauma patients transported to metropolitan hospitals, there were 1166 patients identified as confirmed major trauma at hospital discharge. The current pre-hospital trauma triage criteria have a sensitivity of 95.3%, a specificity of 62.7%, and an overall accuracy of 63.4%. The over-triage rate is 37.3% and the under-triage rate is 4.7%.

Evaluation showed the current triage criteria needed refinement, and multiple revised pre-hospital trauma triage models were constructed. Based on the best overall combination of diagnostic statistics, a revised model was chosen. This updated model includes adjusted blood pressure, respiratory rate and heart rate parameters, removes rollover MVC as a mechanistic consideration, and introduces instructions to transport to a facility within 30 minutes. This model had a sensitivity of 97.8%, a specificity of 82.7% and an accuracy of 83.0%. The over-triage rate was 17.3% and the under-triage rate was 2.2%. The implementation of a revised triage model should identify more confirmed major trauma patients. Likewise, over-triage of non-major trauma patients to major trauma services would be significantly reduced. The refined criteria should also decrease discretionary decision-making by paramedics in the field.

**Davidson GH, Rivara FP, Mack CD, Kaufman R, Jurkovich GJ, Bulger EM. Validation of prehospital trauma triage criteria for motor vehicle collisions. The Journal of Trauma and Acute Care Surgery. 2014;76(3):755-61.**

The American College of Surgeons Committee on Trauma and the Centers for Disease Control jointly developed the National Trauma Triage Protocol: Field Triage Decision Scheme (FTDS), to identify trauma patients who would benefit from trauma center care. The FTDS is based on the stepwise identification of four aspects of clinical presentation including physiologic criteria (PHY), anatomic criteria (ANA), mechanism of injury criteria, and special considerations criteria that are evaluated in a sequential fashion to identify patients who should be transported to a trauma center. Physiologic criteria focus on vital signs and Glasgow Coma Scale; anatomic criteria include specific injury patterns such as penetrating trauma, flail chest, crush injury, etc; mechanism of injury criteria focuses on high risk patterns such as falls from specific height, high speed vehicular crash, motorcycle accident etc; and special considerations include extremes of age, comorbidities, burns, pregnancy, anticoagulated status, etc.

This study sought to determine the likelihood of serious trauma based on vehicle damage sustained in a crash as described in Step 3 of the FTDS. This was a retrospective cross-sectional study including all patients in the United States National Automotive Sampling System Crashworthiness Data System from the years 2003 to 2008. Of 85,761 participants included, 3.7% met step 3 mechanistic criteria alone (having not met step 1 or step 2 criteria). This group had a mean ISS of 5.1 (95% CI, 4.4 - 5.8) and a PPV of 9.7% (95% CI, 9.3 - 10.2%) for severe injury (defined as ISS > 15).

Crash characteristics that predict severe injury included intrusion of greater than 12 inches (PPV of 10.4%; 95% CI, 9.5 - 11.3) and steering wheel collapse (PPV of 25.7%; 95% CI, 23.0 - 28.4%). Older patients (age > 55 years) who met Step 3 mechanism criteria had higher predictive values for injury for nearly all crash characteristics. It was concluded that injury mechanism criteria alone predicted significant injury in a substantial proportion of patients who did not meet the physiologic or anatomic criteria. Vehicular crash data could improve the ability of emergency medical service providers to triage injured occupants. Consideration of transport to a trauma center should be given for elderly patients and drivers with steering wheel collapse.

**Davis JS, Allan BJ, Sobowale O, Ivascu F, Orion K, Schulman CI. Evaluation of a new elderly trauma triage algorithm. Southern Medical Journal. 2012;105(9):447-51.**

Compared with the younger cohort, elderly trauma patients have higher admission rates following injury, longer hospital stays, and higher mortality rates despite lower Injury Severity Scores (ISS). Much of this increased risk of death is the result of pre-existing medical conditions and the effect is greatest in patients with the least severe injuries. Because of these factors, some recommend that the threshold for scene triage or transfer to a trauma center be lowered for elderly adult patients and that failing to do so may be linked to a higher number of unexpected deaths in injured elderly adults. Undertriage is common in patients 55 years and older and is even worse for those 65 and older. In 1999, the Florida legislature implemented a statewide trauma system, including a new Florida trauma triage algorithm (FTTA). The FTTA consists of a series of recommendations based on patient age and abbreviated injury scale scoring of identified injuries, guiding EMS providers to route polytrauma and majorly injured patients to trauma centers. The FTTA includes criteria that are more expansive and was conceived, in part, to combat suspected undertriage. It should be noted that FTTA-positive patients necessarily include all those with an ISS ≥ 16 as well as some ISS 9 to 14 because of specific criteria defined in the FTTA.

This study examined how the new system affected prehospital triage in younger versus older patients. A retrospective chart review of appropriate triage was conducted at the single regional trauma center (level 2) in Polk County, Florida during a one-year period between April 2001 to March 2002. The trauma registry of the trauma center, the hospital discharge dataset of the Florida Agency for Health Care Administration for all hospitals within the county, and Florida Vital Statistics were utilized for data collection. Patients were considered to have major trauma if they were FTTA positive or had an Injury Severity Score (ISS) of ≥16. An internal trauma review panel examined hospital discharge data to assess triage accuracy. A total of 49,726 patients were reviewed of which 2051 were trauma patients. In patients 15 to 54 years old, there was a significant increase in those treated in the trauma center over those treated in the non-trauma center. Eighty-three percent of those considered to be FTTA positive (OR 4.86, 95% CI 3.51 - 6.74) and 86% of those with an ISS ≥ 16 (OR 6.53, 95% CI 4.07- 10.47) were discharged from the trauma center. The same degree of success of the trauma triage criteria was not seen in patients 55 years old and older. In trauma patients in this age group, only 59% of those considered FTTA positive (OR 1.36, 95% CI 1.08 - 2.58) and 64% of those with an ISS ≥ 16 (OR 1.67, 95% CI 1.08- 2.58) were discharged from the trauma center. These percentages are nearly equivalent to the discharge distribution of non-trauma patients, suggesting a minimal effect of the triage criteria in the older age group. The reasons for this finding remain unknown, and further studies are needed to investigate and improve elderly triage.

**Dinh MM, Oliver M, Bein KJ, Roncal S, Byrne CM. Performance of the New South Wales Ambulance Service major trauma transport protocol (T1) at an inner city trauma centre. Emergency Medicine Australasia. 2012;24(4):401-7.**

The New South Wales trauma triage tool is similar to the Field Triage Decision Scheme, with mechanistic, anatomic, and physiologic parameters dictating need for transport to a trauma center in the acronym “MIST”: Mechanism, Injury, Signs and Symptoms, Transport. Mechanism criteria specify markers of high velocity impact such as fall from greater than 3 meters, ejection from vehicle, etcetera; injury criteria focus on a head to toe exam with high risk findings such as altered mentation, neck swelling, bruising from restraints, etcetera; signs and symptoms criteria focus on assessment of airway, breathing circulation and disability parameters. Pregnant women greater than 20 weeks gestation, those on anticoagulants, patients with pre-existing conditions, and pediatric and geriatric populations are highlighted as requiring a high index of suspicion for serious injury.

The study objective was to evaluate the performance of the newly implemented New South Wales prehospital trauma triage (T1) protocol for patients transported to an inner-city major trauma center in Sydney, Australia from March 2010 to October 2011. Ambulances transporting an injured patient on the T1 protocol were required to activate the protocol through an ambulance coordination center and document protocol activation on ambulance case sheets. Upon prehospital notification or arrival to the ED, all patients (whether T1 protocol was activated or not) were screened by the triage nurse using the hospital trauma team activation criteria to determine whether a trauma team response was required.

This was an observational study conducted over 1 year at a single major trauma center. Prehospital data and injury characteristics were collected prospectively for all hospital trauma team activations and injury presentations transported by Ambulance Service of New South Wales. Univariate comparison of T1- and non-T1-transported patients was performed and sensitivity, specificity, over-triage and under-triage rates were calculated. The outcomes studied were Injury Severity Score >15 and major outcome (composite of in-hospital death and/or transferred from the ED to operating theatre or intensive care unit). Factors associated with under-triage were determined with univariate analysis. A total of 2664 ambulance arrivals for trauma were studied with 767 (29%) transported on the T1 protocol. T1-transported patients were associated with more severe injury (23% vs 6%, P < 0.001) and major outcomes (30% vs 10%, P < 0.001) compared with non-T1- transported patients. The sensitivity of the T1 protocol for severe injury was 63% with a positive predictive value of 23%. The under-triage and over-triage rates for severe injury were 12% and 77%, respectively. Under-triaged patients were elderly with falls as the predominant mechanism of injury. The sensitivity and under-triage rates associated with the T1 protocol indicate the ongoing need for secondary triage at designated trauma centers and refinement of the protocol to include age as a criterion.

**Fuller G, Lawrence T, Woodford M, Lecky F. The accuracy of alternative triage rules for identification of significant traumatic brain injury: a diagnostic cohort study. Emergency Medicine Journal. 2014;31(11):914-9.**

Traumatic brain injury (TBI) is a leading cause of death and disability in young adults. Reorganization of trauma services with direct triage of suspected head injury patients to trauma centers may improve outcomes following TBI. This study aimed to determine the sensitivity of principal English triage tools for identifying significant TBI: the London Ambulance Service (LAS) and Head Injury Transportation Straight to Neurosurgery study (HITS-NS) triage criteria. The LAS criteria are similar to the National Trauma Triage Protocol, with physiologic, anatomic and mechanism of injury criteria, along with special considerations (old age, pregnancy, anticoagulated status, morbid obesity), that guide decision making to transport directly to a major trauma center. The main caveat for the LAS criteria is that if a patient has airway obstruction, they should be transported to the closest available hospital for definitive airway management. The HITS-NS rule recommends bypass to a major trauma center for signs of isolated TBI: Glasgow Coma Scale (GCS) ≤13, or evidence of open skull or depressed skull fracture, in the absence of compromised airway, breathing, or circulation. Each triage rule selects contrasting groups of patients for bypass, with the HITS-NS triage rule identifying a higher proportion of isolated TBI patients not requiring immediate ED resuscitation compared with the LAS rule.

The study objective was to evaluate the accuracy of the LAS and HITS-NS triage tools for identifying significant TBI. Distinct objectives were to: estimate the sensitivity and specificity of the LAS and HITS-NS triage rules for significant TBI; describe the characteristics of bypassed, true positive and false negative patients; and examine the relationship between prehospital Glasgow Coma Scale score and anatomical severity of head injury. This study utilized data prospectively collected as part of the English Trauma Audit and Research Network database between 2005 and 2011. Adult head injury patients were retrospectively classified according to the LAS and HITS-NS criteria. Sensitivity and specificity were then calculated against a reference standard of significant TBI, defined as head region abbreviated injury score (AIS) ≥3 or neurosurgical operation. 6559 patients were included in complete case analyses. The LAS and HITS-NS triage tools demonstrated sensitivities of 44.5% (95% CI 43.2 to 45.9) and 32.6% (95% CI 31.4 to 33.9), respectively, and specificities of 69.0% (66.0% to 71.8%) and 89.1% (87.0% to 80.9%) for identifying patients with significant TBI. False negative significant TBI cases were relatively older, more likely to be female, more frequently secondary to low-level falls, and were less likely to have very severe AIS five or six head injuries (p<0.01). Prehospital GCS did not appear to be a sensitive discriminator for identifying significant TBI; 11,794 of the 14,293 study sample patients were classified as having significant TBI with a head region AIS ≥3, of whom 44.9% presented with a prehospital GCS of ≥13. The study concluded that a considerable proportion of significant head injury patients may not be triaged directly to trauma centers. Investment is therefore necessary to improve the accuracy of existing triage rules and maintain expertise in TBI diagnosis and management in non-specialist emergency departments.

**Fuller G, McClelland G, Lawrence T, Russell W, Lecky F. The diagnostic accuracy of the HITSNS prehospital triage rule for identifying patients with significant traumatic brain injury: a cohort study. European Journal of Emergency Medicine. 2016;23(1):61-4.**

Diversion of suspected traumatic brain injury (TBI) patients to trauma centres may improve outcomes by expediting access to specialist neurosurgical care. The HITS-NS rule recommends bypass to a major trauma center for signs of isolated TBI: GCS≤13, or evidence of open skull or depressed skull fracture, in the absence of airway/breathing/circulation compromise.

This study aimed to determine the accuracy of the Head Injury Straight to Neurosurgery (HITS-NS) triage rule for identifying patients with significant TBI. A prospective diagnostic cohort study was performed using data from the HITS-NS trial, the Trauma Audit and Research Network registry and the North East Ambulance service database in the United Kingdom from January 2012 and April 2013. Sensitivity and specificity of the HITS-NS triage rule were calculated against a reference standard of significant TBI, defined by a cranial Abbreviated Injury Scale score of at least 3 or by the performance of a neurosurgical procedure. Eligible cases were coded according to whether they met the triage rule and reference standard criteria, on the basis of their first-recorded prehospital physiology values, clinical parameters and AIS injury codes. Contingency tables (2 × 2) were then constructed to determine true positives, false positives, true negatives and false negatives for the HITSNS triage rule. Prevalence of significant TBI, sensitivity and specificity, with their 95% confidence intervals (95% CIs), were subsequently assessed. A total of 3628 patients were included in the complete case analysis. The HITSNS triage tool demonstrated a sensitivity for severe TBI of 28.3% (95% CI 21.8–35.4) and a specificity of 94.4% (95% CI 93.6–95.2). The low sensitivity of the HITS-NS triage rule suggests that a considerable proportion of patients with significant TBI may not be triaged directly to trauma centers if this triage rule were applied, and further research is needed to improve the accuracy of bypass protocols.

**Gaumont D, Cummins N, Hannigan A, Ryan D. ViEWS from the prehospital perspective: a comparison with a prehospital score to triage categorisation in the emergency department. Irish Medical Journal. 2016;109(6):423.**

The aim of this observational study is to investigate how the recently introduced VitalPac National Early Warning Score (ViEWS) applied in the prehospital setting compares with the Manchester Triage System (MTS) used in most Emergency Departments (ED) in Ireland. To determine ViEWS (similar to the NEWS score), the provider assigns between 0 to 3 points for each of the following vital sign parameters: respiratory rate, oxygen saturation, temperature, systolic blood pressure, heart rate. Additionally, level of consciousness is assigned 0 points if normal, and 3 if abnormal. Any use of supplemental oxygen is assigned 2 points. These points are tallied to form the patient’s ViEWS score. An aggregate score between 0 to 4 is considered low clinical risk; an individual parameter scoring 3, or aggregate score between 5-6 is considered medium clinical risk; an aggregate of 7 or more is considered high clinical risk.

The MTS is a triage algorithm that consists of 52 flowcharts, covering patients’ chief signs and symptoms such as “Headache”, “Shortness of breath” and “Wounds”. Each flowchart in turn consists of additional signs and symptoms named discriminators, such as “Airway compromise”, “Severe pain” or “Persistent vomiting”, which are ranked by priority. Selection of a discriminator allocates the patient to the related urgency category, ranging from “immediate” (0 minutes maximum waiting time) to “non-urgent” (240 minutes maximum waiting time). Level 1 is classified as an emergency and level 5 as nonurgent.

It was hypothesized that most patients triaged with a MTS of 1 or 2 would have a high ViEWS score of ≥7. This was a retrospective observational study conducted at a single center in Ireland over a period of 6 months. 386 patients met the inclusion criteria of which 272 (69 %) had a complete set of values. Of 272 MTS 1 & 2 patients, only 114 (42%) had a Ph-ViEWS ≥7. This study found that a substantial number of patients deemed urgent at the time of triage do not have elevated prehospital ViEWS.

**Giannakopoulos GF, Saltzherr TP, Lubbers WD, Christiaans HM, van Exter P, de Lange-de Klerk ES, et al. Is a maximum Revised Trauma Score a safe triage tool for Helicopter Emergency Medical Services cancellations? European Journal of Emergency Medicine. 2011;18(4):197-201.**

The Revised Trauma Score is used worldwide in the prehospital setting and provides a snapshot of the patient’s physiological state. In its current, commonly used form, the RTS ranges between 0 and 12 and is calculated from three physiologic parameters: the Glasgow Coma Scale (GCS), respiratory rate, and systolic blood pressure, which are weighted to compute a final score. A score of 12 suggests a physiologically stable patient. In the Netherlands, Helicopter Emergency Medical Services (HEMS) are mostly used for delivery of specialized trauma teams on scene and occasionally for patient transportation. In the Dutch trauma system, the Emergency Medical Services crew performs triage after arrival on scene and cancels the HEMS-dispatch if deemed unnecessary.

This retrospective study examined the ability of a maximum on-scene Revised Trauma Score (RTS = 12) to be used as a triage tool for HEMS cancellation. All patients with a maximum on-scene RTS after blunt trauma (with or without receiving HEMS care) who presented to the trauma resuscitation room of two Level-1 trauma centers during a period of 6 months were included. Information concerning prehospital and in-hospital vital parameters, severity and localization of the injuries, and the in-hospital course were analyzed. Major trauma patients were classified using the following parameters: Injury Severity Score of at least 16, emergency intervention, Intensive Care Unit admission, and in-hospital death. 440 blunt trauma patients having a maximum RTS were included between 1 July and 31 December 2006. 80 patients received on-scene HEMS care. Almost 16% (n = 67) of the total study population consisted of major trauma patients, of which only 25 (36%) received HEMS care. In 17 patients (3.9%), the RTS deteriorated during transportation. Major trauma patients sustained more injuries to the chest, abdomen, and lower extremities. The authors concluded that the RTS alone is not a reliable triage tool for HEMS cancellations in the Dutch trauma system and will lead to a considerable rate of under-triage with one in every six cancellations being incorrect. Other criteria based on patient’s vital signs, combined with anatomical and mechanism of injury parameters should be developed to safely minimize triage errors.

**Helwig SA, Ragoschke-Schumm A, Schwindling L, Kettner M, Roumia S, Kulikovski J, et al. Prehospital Stroke Management Optimized by Use of Clinical Scoring vs Mobile Stroke Unit for Triage of Patients With Stroke: A Randomized Clinical Trial. JAMA Neurology. 2019;03:03.**

The Los Angeles Motor Scale (LAMS) is a brief 3-item scale that focuses only on motor symptoms. It involves assessment of facial droop (0-1 point), arm drift (0-2 points) and grip strength (0-2 points) and patients with a score of 4 or greater are considered to be high risk for large vessel occlusion.

The objective of this study was to determine how optimized prehospital management (OPM) based on use of the Los Angeles Motor Scale (LAMS) compares with management in a Mobile Stroke Unit (MSU) in accurately triaging patients to the appropriate hospital with or without interventional treatment (comprehensive vs. primary stroke center). In this randomized multicenter trial with 3-month follow-up, patients were assigned week-wise to one of the pathways between June 2015 and November 2017, in 2 regions of Saarland, Germany; 708 of 824 suspected stroke patients did not meet inclusion criteria, resulting in a study population of 116 adult patients.

Patients received either OPM based on a standard operating procedure that included the use of the LAMS (cut point 4) or management in an MSU (an ambulance with vascular imaging, point-of-care laboratory, and telecommunication capabilities). The MSU intervention consisted of neurologic examination, POC testing, and non-contrast CT, as well as CT angiography for patients with no ICH. The primary endpoint was the proportion of patients accurately triaged to either CSCs (LVO, ICH) or PSCs (others). A predefined interim analysis was performed after 116 patients of the planned 232 patients had been enrolled. Of these, 53 were included in the OPM group (67.9% women; mean [SD] age, 74 [11] years) and 63 in the MSU group (57.1% women; mean [SD] age, 75 [11] years). The primary end point, an accurate triage decision, was reached for 37 of 53 patients (69.8%) in the OPM group and for 63 of 63 patients (100%) in the MSU group (difference, 30.2%; 95% CI, 17.8%-42.5%; P < .001).

Whereas 7 of 17 OPM patients (41.2%) with LVO or ICH required secondary transfers from a PSC to a CSC, none of the 11 MSU patients (0%) required such transfers (difference, 41.2%; 95% CI, 17.8%-64.6%; P = .02). The LAMS at a cut point of 4 or higher led to an accurate diagnosis of LVO or ICH for 13 of 17 patients (76.5%; 6 triaged to a CSC) and of LVO selectively for 7 of 9 patients (77.8%; 2 triaged to a CSC). Stroke management metrics were better in the MSU group, although patient outcomes were not significantly different. Whereas prehospital management optimized by LAMS allows accurate triage decisions for approximately 70% of patients, MSU-based management enables accurate triage decisions for 100%. Depending on the specific health care environment considered, both approaches are potentially valuable in triaging stroke patients.

**Hoikka M, Silfvast T, Ala-Kokko TI. Does the prehospital National Early Warning Score predict the short-term mortality of unselected emergency patients? Scandinavian Journal of Trauma, Resuscitation and Emergency Medicine. 2018;26(1):48.**

The National Early Warning Score (NEWS) was originally designed to detect deteriorating patients in hospital wards, specifically those at increased risk of ICU admission, cardiac arrest, or death within 24 hours. To determine NEWS, the provider assigns between 0 to 3 points for each of the following vital sign parameters: respiratory rate, oxygen saturation, temperature, systolic blood pressure, heart rate. Additionally, level of consciousness is assigned 0 points if normal, and 3 if abnormal. Any use of supplemental oxygen is assigned 2 points. These points are tallied to form the patient’s NEWS score. An aggregate score between 0 to 4 is considered low clinical risk; an individual parameter scoring 3, or aggregate score between 5-6 is considered medium clinical risk; an aggregate of 7 or more is considered high clinical risk.

The use of NEWS in the prehospital setting may facilitate earlier identification of patients at risk. However, in daily practice the value of risk assessment to support decision-making in prehospital setting is unclear. This study aimed to examine the accuracy of the prehospitally implemented NEWS in predicting 1-day and 30-day mortalities in an unselected EMS population. A secondary aim was to describe the causes of death in this prehospital patient population.

Data from all emergency medical service (EMS) situations were coupled to the mortality data obtained from the Causes of Death Registry during a six-month period in 2014 in Northern Finland. NEWS values were calculated post hoc from first clinical parameters obtained on the scene and patients were categorized to the low, medium and high-risk groups accordingly. Sensitivities, specificities, positive predictive values (PPVs), negative predictive values (NPVs), and likelihood ratios (PLRs and NLRs) were calculated for 1-day and 30-day mortalities at the cut-off risks. A total of 12,426 EMS calls were included in the study. The overall 1-day and 30-day mortalities were 1.5 and 4.3%, respectively. The 1-day mortality rate for NEWS values ≤12 was lower than 7% and for values ≥13 higher than 20%. The high-risk NEWS group had sensitivities for 1-day and 30-day mortalities of 0.801 (CI 0.74–0.86) and 0.42 (CI 0.38–0.47), respectively. In the prehospital environment, the high-risk NEWS category was associated with 1-day mortality well above that of the medium and low risk NEWS categories. This effect was not as noticeable for 30-day mortality. The authors conclude that use of NEWS in the prehospital setting may be of value when assessing the mortality risk within 24 hours and hence immediate need for medical care. However, based on this cohort, they conclude that NEWS alone cannot guide decision-making about the urgency of transport, the destination of transport, or whether to transport or not, and needed to be further studied.

**Johansson N, Spindler C, Valik J, Vicente V. Developing a decision support system for patients with severe infection conditions in pre-hospital care. International Journal of Infectious Diseases. 2018;72:40-8.**

The objective of this study was to develop and validate a pre-hospital decision support system (DSS) for the emergency medical services (EMS) in Sweden, enabling the identification and steering of patients with critical infectious conditions (i.e., severe respiratory tract infections, severe central nervous system (CNS) infections, and sepsis) to a specialized emergency department (ED) for infectious diseases. The development process involved four consecutive steps. The first step was gathering data from the electronic patient care record system (ePCR) on patients transported by the EMS, in order to identify retrospectively appropriate patient categories for steering. The second step was to let a group of medical experts give advice and suggestions for further development of the DSS. The third and fourth steps were the evaluation and validation, respectively, of the whole pre-hospital DSS in a pilot study.

The pilot study involved pre-hospital emergency nurses (PENS) from a single EMS agency in Sweden. The study included 72 patients, of whom 60% were triaged to a highly specialized emergency department (ED-Spec) with an attending infectious disease physician (ID physician). The results demonstrated that the PENs adhered to the DSS in 66 of 72 patient cases (91.6%). For those patients steered to the ED-Spec, the assessment made by PENs and the ID physician at the ED was concordant in 94% of cases. It was concluded that the development of a specific DSS aiming to identify patients with three different severe infectious diseases appears to give accurate decision support to PENs when steering patients to the optimal level of care.

The DSS for respiratory infection is shared within the manuscript, but the DSS for CNS infection and sepsis are not. The respiratory infection DSS specifies that any patient with relatively stable vitals, or extremely unstable vitals have to be triaged to the closest emergency department, whereas those with moderately unstable vitals should be triaged to the ED-Spec.

**Jumaa MA, Castonguay AC, Salahuddin H, Shawver J, Saju L, Burgess R, et al. Long-term implementation of a prehospital severity scale for EMS triage of acute stroke: a real-world experience. Journal of Neurointerventional Surgery. 2019;02:02.**

The Rapid Arterial oCclusion Evaluation (RACE) Scale is one of the few prehospital scales that have been prospectively validated in the field. Patients testing positive for suspected stroke on the Cincinnati Stroke Scale are subsequently assessed by EMS using the RACE Scale. The RACE scale evaluates five items: facial palsy, upper extremity paresis, lower extremity paresis, head and gaze deviation, and aphasia/agnosia, with a total score of 0–9. Patients with a RACE score ≥5 and last seen normal within 24 hours or time of onset unknown are triaged as RACE-alerts (RA) and sent to the nearest ECC. Patients with a RACE score <5 are triaged as stroke-alerts (SA) and transported to the nearest hospital.

The study objective was to report the long-term experience of a US countywide emergency medical services (EMS) acute stroke triage protocol using the RACE score. A prospective database was used to identify all consecutive patients triaged within Lucas County, Ohio by the EMS with (1) a RACE score ≥5, taken directly to an endovascular capable center (ECC) as RACE-alerts (RA) and (2) a RACE score <5, taken to the nearest hospital as stroke-alerts (SA). Baseline demographics, RACE score, time metrics, final diagnosis, treatments, and clinical and angiographic outcomes were captured. The sensitivity and specificity for patients with a RACE score ≥5 with LVO, eligible for mechanical thrombectomy (MT), were calculated. Between July 2015 and June 2018, 492 RA and 1147 SA were triaged within this five-hospital network. Of the RA, 37% had AIS secondary to LVOs. Of the 492 RA and 1147 SA, 125 (25.4%) and 38 (3.3%), respectively, underwent MT (OR=9.9; 95% CI 6.8 to 14.6; p<0.0001). Median times from onset-to­ECC arrival (74 vs 167min, p=0.03) and dispatch-to­ECC arrival (31 vs 46 min, p=0.0002) were shorter in the RA-MT than in the SA-MT cohort. A RACE cut-off point ≥5 showed a sensitivity and specificity of 77% and 75% for detection of patients with LVO eligible for MT, respectively. This study demonstrates the long-term feasibility of a countywide EMS-based prehospital triage protocol using the RACE Scale.

**Kesinger MR, Sequeira DJ, Buffalini S, Guyette FX. Comparing National Institutes of Health Stroke Scale among a stroke team and helicopter emergency medical service providers. Stroke. 2015;46(2):575-8.**

The National Institutes of Health Stroke Scale (NIHSS) has been traditionally used in the in-hospital setting to quantify stroke severity. Providers calculate a score ranging from 0 to 42 based on the following parameters: level of consciousness, orientation, ability to follow motor commands, horizontal gaze, visual fields, facial palsy, motor drift of all extremities, limb ataxia, sensation, language/aphasia, dysarthria, and extinction/inattention. A NIHSS score ≥12 is predictive of large vessel occlusion (LVO).

The objective of this study was to evaluate whether prehospital providers could reliably calculate NIHSS. The authors compared helicopter emergency medical services (HEMS) providers’ calculated NIHSS compared with in-hospital stroke team physician scores for all patients with ischemic stroke transported by HEMS to a single comprehensive stroke center in Pennsylvania, USA, in 2010. HEMS NIHSS were compared with in-hospital stroke team physician scores and ability to predict LVO was investigated.

Three-hundred five patients met inclusion criteria, with 68.9% having LVO. Moderate agreement existed between HEMS and physicians (72.1%; κ=0.571). Interclass correlation was 0.879 (95% confidence interval, 0.849–0.904). Excluding patients with tissue-type plasminogen activator before HEMS transport, there were 216 patients and good agreement (82.7%; κ=0.619). Among patients presenting within 8 hours post-onset and NIHSS≥12, HEMS had a sensitivity of 55.9% and positive predictive value of 83.7% in predicting LVO. It was concluded that HEMS providers can administer NIHSS with moderate to good agreement with the receiving stroke team. The use of the NIHSS in HEMS may identify patients with LVO and inform triage decisions for patients’ ineligible for tissue-type plasminogen activator.

**Lavoie A, Emond M, Moore L, Camden S, Liberman M. Evaluation of the Prehospital Index, presence of high-velocity impact and judgment of emergency medical technicians as criteria for trauma triage. CJEM Canadian Journal of Emergency Medical Care. 2010;12(2):111-8.**

The Prehospital Index (PHI) consists of 5 elements observed on scene: systolic blood pressure, heart rate, respiratory status, level of consciousness and penetrating torso injury. It increases with severity and ranges from 0 to 24. A score of 4 serves as the cut-off point for transport to a trauma center. The evaluation of high velocity impact (HVI) presence is intended to identify injuries involving a high level of energy transfer. Specific examples include a fall greater than 7 m (20 ft), another victim killed in the same accident, ejection from a motor vehicle, cabin structural intrusion and pedestrian or cyclist hit by a motor vehicle travelling faster than 8 km/h (5 mile/h).

The objective of this study was to evaluate the performance of the PHI, the HVI criterion and emergency medical technician (EMT) judgment for the prehospital triage of injured patients. This study retrospectively evaluated the validity of 3 widely used trauma triage criteria on a consecutive series of adult patients served by 2 level-I urban trauma centers in Montreal, Canada. All consecutive trauma patients who were treated on scene by EMTs and directly transported to one of the 2 designated level-I trauma centers between Jan. 1, 1997, and Dec. 31, 2000, were identified and included if they met criteria. The main outcome was severe trauma, defined as death within 72 hours, admission to the intensive care unit within 24 hours or an Injury Severity Score greater than 15. The sensitivity, specificity and rates of overtriage (1 – positive predictive value) for each criterion and their combinations were assessed.

Of 16,805 patients in the study population, 1113 (6.62%) had severe trauma. The combination of all 3 triage criteria (PHI score ≥ 4, HVI presence and EMT judgment) performed best for identifying patients with severe trauma, with a sensitivity of 74.2%, specificity 70.0%, positive predictive value 14.9% and negative predictive value of 98.2%, with an overtriage rate of 85.1%. Alone, EMT judgment had the highest sensitivity and a PHI score of 4 or greater had the lowest rate of overtriage. Although the combination of PHI score, HVI presence and EMT judgment offers the highest sensitivity for the identification of patients that could benefit from direct transport to a level-I trauma centre, overall sensitivity remains low and overtriage is high. More research is required to improve prehospital triage.

**Leeies M, Ffrench C, Strome T, Weldon E, Bullard M, Grierson R. Prehospital Application of the Canadian Triage and Acuity Scale by Emergency Medical Services. CJEM Canadian Journal of Emergency Medical Care. 2017;19(1):26-31.**

The Canadian Triage and Acuity Scale is a validated five-level triage score utilized in Emergency Departments (EDs) across Canada and internationally. The CTAS consists of five triage levels combining severity of illness or risk with recommended fractile response times to medical assessment supported by a standardized patient presenting complaint list. Level 1 is considered most urgent and Level 5 least urgent. The objective of this study is to prospectively evaluate CTAS interrater reliability between emergency medical services (EMS) providers and ED triage nurses with a system-wide implementation during routine clinical practice. This was a prospective, observational cohort study of all patients ≥17 years old transported to any ED in the Winnipeg Region, Canada by Winnipeg Fire and Paramedic Service (WFPS) EMS from July 1, 2014–December 31, 2014. Variables were extracted from hospital and EMS databases. The primary outcome was interrater reliability for triage nurse and EMS CTAS scores. The hospital arrival EMS CTAS (CTAS-arrival) score was compared to the initial nursing CTAS score (CTAS-initial) and the final nursing CTAS score (CTAS-final) including nursing overrides. Interrater reliability between ED CTAS-initial and EMS CTAS-arrival scores was assessed. Interrater reliability between ED CTAS-final and EMS CTAS-arrival scores, as well as proportion of patient encounters with perfect or near-perfect agreement, were evaluated.

14,378 consecutive adult patient encounters in the Winnipeg Region were evaluated. It was observed that, when compared to the triage nurses, EMS tended to score patients equal or lower for CTAS 1, 2, and 3 patients, but tended to score equal or higher for CTAS 4 and 5 patients. The primary outcome, interrater reliability between EMS CTAS arrival and ED CTAS initial scores (the CTAS score prior to overrides), indicated moderate agreement [κw = 0.437 (p<0.001, 95% CI 0.421-0.452)]. EMS CTAS-arrival and ED CTAS-initial scores had an exact or within one-point match 84.3% of the time. The secondary interrater reliability outcome between EMS CTAS-arrival and ED CTAS-final score (including nurse overrides) also showed moderate agreement [κw = 0.452 (p<0.001, 95% CI 0.437-0.466)] and an exact or within one-point match 86.5% of the time. Further research is needed to define the role of CTAS in EMS systems where alternate levels of care and/or delayed- or non-transport processes are incorporated.

**Lerner EB, Cushman JT, Drendel AL, Badawy M, Shah MN, Guse CE, et al. Effect of the 2011 Revisions to the Field Triage Guidelines on Under- and Over-Triage Rates for Pediatric Trauma Patients. Prehospital Emergency Care. 2017;21(4):456-60.**

The Field Triage Decision Scheme was developed and first published by The American College of Surgeons – Committee on Trauma (ACS-COT) in 1987. This initial guideline laid the foundation for EMS providers to recognize patients who would benefit from transport to a specialized trauma center. After its initial publication in 1986, the American College of Surgeons Committee on Trauma updated the Field Triage Decision Scheme in 1990, 1993, and 1999. In 2005, the ACS-COT collaborated with the Centers for Disease Control and Prevention (CDC) to form a National Expert Panel on Field Triage. The objective of the study was to determine the change in under- and over-triage rates when the 2011 Field Triage Guidelines are compared to the 2006 and 1999 versions. A three-year prospective cohort study was conducted in 3 pediatric hospitals that also served as the regional pediatric trauma center located in Dallas, Texas, Milwaukee, Wisconsin, and Rochester, New York, United States. Pediatric patients were defined as those less than 15 years old. The 1999, 2006, and 2011 Field Triage Guidelines were each retrospectively applied to the collected data and descriptive statistics were used to compare between Guideline versions.

Applying the 1999, 2006, or 2011 Guidelines to the EMS interview data the over-triage rate was 32.6%, 27.9%, and 28.0%, respectively. The under-triage rate was 26.5%, 35.1%, and 34.8%, respectively. The 2011 Guidelines resulted in an 8.2% (95% CI 0.6–15.9%) absolute increase in under-triage and a 4.6% (95% CI 2.8–6.3%) decrease in over-triage compared to 1999 Guidelines.

**Leung SC, Leung LP, Fan KL, Yip WL. Can prehospital Modified Early Warning Score identify non-trauma patients requiring life-saving intervention in the emergency department? Emergency Medicine Australasia. 2016;28(1):84-9.**

The prehospital Modified Early Warning Score (MEWS) was first validated by Subbe *et al.* in 2001 in a medical admission unit and was thereafter reported to be useful in identifying those at risk among acute medical patients, surgical in-patients and those requiring inter-facility transfer. It may also be a good predictor of need for acute hospitalisation and adverse in-hospital outcomes. It is calculated from the first set of clinical observation data and physiological parameters recorded by ambulance crews. MEWS comprises the alert, voice, pain, unresponsive (AVPU) score and four physiological domains: systolic blood pressure, heart rate, respiratory rate and body temperature. The overall MEWS is calculated to produce a score ranging between 0 and 14. The objective of this study was to investigate whether prehospital MEWS could predict the need for life-saving intervention (LSI) within 4 h of ED presentation in ambulance patients. This was a prospective study of non-trauma ambulance patients who were 16 years or older and treated in the ED from 1 to 27 November 2013 in a university-teaching hospital in Hong Kong. Data on patients’ demographics, triage category, LSI within 4 h of ED presentation and 24 h mortality were retrieved.

Regarding the need for LSI, the sensitivity, specificity, predictive values and likelihood ratios of prehospital MEWS were calculated. A score equal to or greater than a chosen cut-off point suggested an indication to pre-alert the receiving ED. The overall performance of MEWS was assessed with the area under the receiver operating characteristic curve (AUC). Of 1493 recruited patients, 321 (21.5%) required LSI within 4 h of ED presentation. Recruited patients’ prehospital MEWS ranged from 0 to 11. The distribution of scores was positively skewed, and the median MEWS was 2 (interquartile range, 1–3). Both LSI implementation and mortality increased with MEWS in general. The median MEWS to predict at least one mode of LSI was 4 or above. All cases required LSI among those with MEWS ≥9. There was no mortality among cases with MEWS ≥10. True and false positive rates of LSIs with 95% CI were calculated for each cut-off point of MEWS for the construction of a ROC curve. The AUC was 0.72 (95% CI 0.69 to 0.75), which indicated a fair discrimination between those with or without LSIs. The sensitivity, specificity, positive PV, negative PV, positive LR and negative LR were 0.57, 0.76, 0.40, 0.87, 2.43 and 0.56, respectively, when prehospital MEWS ≥3 was chosen as the cut-off value.

In conclusion, prehospital MEWS may perhaps play a role in predicting the need for LSI within 4 h of ED presentation, with a trade-off between number of cases to pre-alert the receiving ED and sensitivity of detection. Its addition to any ED pre-alert or triage protocol should be considered. Future validation work with a larger sample size in multicentre settings is advocated, in comparison with clinical decisions by staff with variable years of experience, to substantiate the use of prehospital MEWS to predict LSI and short-term mortality.

**Li JL, McMullan JT, Sucharew H, Broderick JP, Katz B, Schmit P, et al. Potential Impact of C-STAT for Prehospital Stroke Triage up to 24 Hours on a Regional Stroke System. Prehospital Emergency Care. 2019:1-5.**

The Cincinnati Stroke Triage Assessment Tool (CSTAT) is a prehospital tool intended to be applied in suspected stroke patients to screen for severe versus non-severe stroke. It comprises of Conjugate Gaze Deviation (2 points), Incorrectly answering age/month AND not following at least one command (close your eyes, open and close your hand) (1 point), and Arm (right, left, or both) falling to bed within 10 seconds (1 point). Having a score of 2 or greater equals a high likelihood of large vessel occlusion (LVO) stroke.

The objective of this study was to estimate the potential impact of the expanded thrombectomy time window on suspected AIS-LVO cases transported to the regional comprehensive stroke center (CSC). The study population consisted of all patients transported to any urban hospital by the Cincinnati Fire Department with a prehospital clinical impression of stroke or transient ischemic attack (TIA) from June 1 to November 30, 2015. Patients 18 years or older were included in the data analysis if they were both CPSS positive by EMS and had a calculable C-STAT, and had outcome data in their medical records. For this analysis, the outcome was the proportion of patients with a suspected stroke/TIA and Last Known Normal of 0–6 or 6–24 hours that would be transported to a CSC with prehospital C-STAT-based triage. C-STAT test characteristics, sensitivity and specificity, for LVO (defined as ICA and M1 occlusions only) were calculated with 95% confidence intervals (CI) using the Wilson interval.

Of 158 patients with prehospital suspicion for stroke/TIA, 105 were CPSS positive within 24 hours of onset and had complete C-STAT and clinical data available for analysis. C-STAT sensitivity and specificity for LVO were 71% (95% CI 36–92) and 67% (95% CI 58–80), respectively. C-STAT triage would increase transport of prehospital suspected stroke cases to the CSC by 11% (12/105) within six hours and 21% (22/105) within 24 hours. Of 37 C-STAT positive patients, only 5 (13.5%) had LVO as final diagnosis. Preferential triage of prehospital suspected stroke patients using C-STAT would increase the number of patients transported to the CSC by 11% within six hours and an additional 10% from six to 24 hours. For every patient with LVO as final diagnosis, approximately an additional 6 non-LVO patients would be triaged to a comprehensive stroke center.

**Lima FO, Silva GS, Furie KL, Frankel MR, Lev MH, Camargo EC, et al. Field Assessment Stroke Triage for Emergency Destination: A Simple and Accurate Prehospital Scale to Detect Large Vessel Occlusion Strokes. Stroke. 2016;47(8):1997-2002.**

The FAST-ED scale (Facial Palsy (scored 0–1), Arm Weakness (0–2), Speech Changes (0– 2), Time (documentation for decision making but no points), Eye Deviation (0-2), and Denial/Neglect (0-2)) was designed based on items of the NIHSS with higher predictive value for large vessel occlusion stroke (LVOS). In addition, time was included considering its importance in the prehospital decision algorithm. The objective of this study was to evaluate performance of the Field Assessment Stroke Triage for Emergency Destination (FAST-ED) tool for LVO detection. The scale was tested on data from 741 consecutive patients enrolled in a prospective cohort study at two university-based hospitals (STOPStroke cohort), in California, USA, in which patients underwent CT angiography within the first 24 hours of stroke onset.

Receiver operating characteristic (ROC) curve, sensitivity, specificity, positive (PPV) and negative predictive values (NPV) of FAST-ED were compared with the NIHSS, Rapid Arterial occlusion Evaluation (RACE) scale and Cincinnati Prehospital Stroke Severity Scale (CPSSS). LVO was detected in 240 of the 727 qualifying patients (33%). The FAST-ED scale had comparable accuracy to predict LVO to the more complex NIHSS and higher accuracy than RACE and CPSS (area under the ROC curve: FAST-ED = 0.81 as reference; NIHSS = 0.80, p = 0.28; RACE = 0.77, p = 0.02; and CPSS = 0.75, p = 0.002). A FAST-ED ≥4 had sensitivity of 0.60, specificity 0.89, PPV 0.72, and NPV 0.82 versus RACE ≥5 of 0.55, 0.87, 0.68, 0.79 and CPSS ≥2 of 0.56, 0.85, 0.65, 0.78, respectively. FAST-ED is a simple scale that if successfully validated in field might be useful for medical emergency professionals to accurately identify LVOS in the prehospital setting enabling rapid triage of patients to primary versus endovascular capable stroke centers.

**Magnusson C, Herlitz J, Karlsson T, Axelsson C. Initial assessment, level of care and outcome among children who were seen by emergency medical services: a prospective observational study. Scandinavian Journal of Trauma, Resuscitation and Emergency Medicine. 2018;26(1):88.**

The assessment of children in the Emergency Medical Service (EMS) is infrequent representing 5.4% of the patients in an urban area in the western part of Sweden. In Sweden, patients are assessed on scene by an EMS nurse whom independently decides on interventions and level of care. To aid the EMS nurse in the assessment a triage instrument, Rapid Emergency Triage and Treatment System-pediatrics (RETTS-p) developed for Emergency Department (ED) purpose has been in use the last 5 years.

The Rapid Emergency Triage and Treatment System-pediatrics (RETTS-p) was initially developed for triaging within the pediatric Emergency Department (pED). The RETTS-p is made up of two parts, vital signs (VS) and emergency signs and symptoms (ESS). The level of severity is based on the highest color of ESS or VS that becomes the final triage level. Red is stated as life threatening, orange is potentially life threatening and both levels are, from a time perspective, defined as emergency care directly. Yellow and green are defined as no individual medical risk if put on wait for assessment by a physician. Five VS, respiratory rate, oxygen saturation, pulse frequency, body temperature and level of consciousness, are recorded and adjusted to age intervals, including pulse correction for patients presenting with fever.

The aim of this study was to evaluate the EMS nurse assessment, management, the utilization of RETTS-p and patient outcome. A prospective, observational study was performed on 651 children aged < 16 years from January to December 2016. Statistical tests used in the study were Mann-Whitney U test, Fisher’s exact test and Spearman’s rank statistics.

The dispatch centre indexed life-threatening priority in 69% of the missions but, of all children, only 6.1% were given a life threatening RETTS-p red colour by the EMS nurse. A total of 69.7% of the children were transported to the ED and, of these, 31.7% were discharged without any interventions. Among the non-conveyed patients, 16 of 197 (8.1%) visited the ED within 72 h but only two were hospitalised. Full triage, including five out of five vital signs measurements and an emergency severity index, was conducted in 37.6% of all children. A triage colour was not present in 146 children (22.4%), of which the majority were non-conveyed. The overall 30-day mortality rate was 0.8% (n = 5) in children 0–15 years. Despite the incomplete use of all vital signs according to the RETTS-p, the EMS nurse assessment of children appears to be adapted to the clinical situation in most cases and the patients appear to be assessed to the appropriate level of care but indicating an over triage. It seems that the RETTS-p with full triage is used selectively in the pre-hospital assessment of children with a risk of death during the first 30 days of less than 1%.

**Magnusson C, Herlitz J, Karlsson T, Jimenez-Herrera M, Axelsson C. The performance of the EMS triage (RETTS-p) and the agreement between the field assessment and final hospital diagnosis: a prospective observational study among children < 16 years. BMC Pediatrics. 2019;19(1):500.**

The Rapid Emergency Triage and Treatment System-pediatrics (RETTS-p) was initially developed for triaging within the pediatric Emergency Department (pED) and has been used by the emergency medical services (EMS) in the west of Sweden since 2014. The RETTS-p is made up of two parts, vital signs (VS) and emergency signs and symptoms (ESS). Five VS, respiratory rate, oxygen saturation, pulse frequency, body temperature and level of consciousness, are recorded and adjusted to age intervals, including pulse correction for patients presenting with fever. The level of severity is based on the highest color of ESS or VS that becomes the final triage level. Red is stated as life threatening, orange is potentially life threatening and both levels are, from a time perspective, defined as emergency care directly. Yellow and green are defined as no individual medical risk if put on wait for assessment by a physician. The aim of this study was to evaluate the performance of the RETTS-p in the EMS and the agreement between the EMS field assessment and the hospital diagnosis.

This study is an observational prospective study of pediatric patients assessed by an EMS nurse and transported to the pediatric Emergency Department in a single-site urban setting in the western part of Sweden. All children below 16 years of age in this sample were included if the inclusion criteria were met. A total of 716 children were initially identified in the sample and 65 children were excluded as they did not fulfil the inclusion criteria. Positive and negative predicted values and likelihood ratios from data were used to calculate sensitivity and specificity. Under-triage and over-triage was defined as 1-Sensitivity (proportion of yellow/green triaged among all emergent patients) and 1-Specificity (proportion of red/orange triaged among all nonemergent patients) respectively. If triaged to yellow or green, the predictive value of not being life threatening or potentially life threatening was 91.7, 95% CI [88.5,94.1]. The RETTS-p triage levels of red and orange had a sensitivity of 66.7% for detecting an emergent patient, 95% CI [53.3,78.3], i.e. an under-triage of 33.3%. The corresponding specificity was 67.0, 95% CI [61.6,72.0], i.e. an over-triage of 33.0%. The positive likelihood ratio was 2.02, 95% CI [1.59, 2.56], and the negative likelihood ratio was 0.47, 95% CI [0.33, 0.67]. The EMS field assessment agreed with the final hospital diagnosis in 80% of the cases.

The RETTS-p sensitivity in this study is considered moderate. Two thirds of the children triaged to life threatening or potentially life threatening were later identified as non-emergent. Of those, one in six was discharged from the PED without any intervention. Further, one third of the children were under triaged, the majority were found in the yellow triage level (can wait). The highest proportion of hospitalised patients was found in the yellow triage level. The result agreed with previous studies using other triage instruments. A computerized decision support system might help the EMS triage to increase sensitivity and specificity.

**Martin-Rodriguez F, Lopez-Izquierdo R, Del Pozo Vegas C, Delgado-Benito JF, Del Pozo Perez C, Carbajosa Rodriguez V, et al. A Multicenter Observational Prospective Cohort Study of Association of the Prehospital National Early Warning Score 2 and Hospital Triage with Early Mortality. Emergency Medicine International Print. 2019;2019:5147808.**

The National Early Warning Score 2 (NEWS2) is the most used internationally and validated scale for assessment of patient acuity in the prehospital context. The clinical variables for the NEWS2 are: respiratory rate, oxygen saturation, heart rate, systolic blood pressure, temperature, confusion state (confusion was defined as a score of less than 15 points on the Glasgow Coma Scale), and the use of oxygen.

The aim of this study was to evaluate the capacity of the prehospital scale NEWS2 in relation to the classification of Spanish hospital triage to predict early mortality within 48 hours of the index event. In addition, the performance of this scale in estimating mortality at 7 and 30 days was explored. A prospective multicenter longitudinal observational cohort study was carried out in three provinces of Spain on all patients cared for by the Advanced Life Support (ALS) system. Patients were included in the study if they had been evaluated and transferred by an ALS to the ED of the reference hospital and did not meet any exclusion criteria. Between April 1 and November 30, 2018, a total of 1054 patients were included in the study. The predictive power of the NEWS2 scale to discriminate mortality at 2, 7, and 30 days is evidenced by an AUC of 0.88 (95% CI: 0.82-0.94), 0.86 (95% CI: 0.81-0.91), and 0.82 (95% CI: 0.77-0.87). In the stratification by the Spanish five level triage system, the AUC of the NEWS2 obtained for short-term mortality varied between 0.77 (95% CI: 0.65-0.89) for level I (resuscitation) and 0.94 (95% CI: 0.79-1) for level III (urgent). In the analysis of short-term mortality, it was observed that a NEWS2 score greater than or equal to 7 among patients with priority III had a sensitivity of 100 (95% CI 56.6-100) and a specificity of 78.7 (95% CI 75.1-81.9) with a PPV of 4.1 (1.8-9.3) and a NPV of 100 (99.1- 100). Meanwhile, in patients with priorities I and II, the cutoff point with better sensitivity and joint specificity rose to 9 points in both cases, with associated NPV of 92.5 (95% CI: 82.1-97.0) for level I and 98.4 (95% IC: 96.2-99.3) for level II. The Prehospital Emergency Medical Services should evaluate the implementation of the NEWS2 as a routine evaluation, which, together with the structured hospital triage system, effectively serves to predict early mortality and detect high-risk patients.

**McMullan JT, Katz B, Broderick J, Schmit P, Sucharew H, Adeoye O. Prospective Prehospital Evaluation of the Cincinnati Stroke Triage Assessment Tool. Prehospital Emergency Care. 2017;21(4):481-8.**

FAST (also known as the Cincinnati Prehospital Stroke Scale) and C-STAT assessments were performed by EMS personnel at the time of initial patient evaluation. FAST consists of three elements: facial droop, arm drift, and speech abnormality (dysarthria or aphasia). The C-STAT is comprised of select NIHSS items (1b and 1c—level of consciousness commands and questions, 2—gaze, and 5—arm motor) and simplified to either normal or abnormal. The objective of this study is to describe the feasibility of prehospital implementation of the C-STAT to identify subjects with severe stroke (NIHSS ≥ 15) among all prehospital patients with clinical suspicion of stroke. Secondarily, the ability for the tool to identify subjects with NIHSS ≥10, the presence of LVO, or the need for services available only at a comprehensive stroke center (CSC) was evaluated.

Over a period of six months (June–November 2015), all patients transported by Cincinnati Fire Department with a prehospital clinical impression of stroke or transient ischemic attack (TIA) were screened with FAST and evaluated with C-STAT. C-STAT performance among FAST positive subjects was assessed for predicting stroke severity (NIHSS15, NIHSS10), LVO, and CSC need. Sensitivity, specificity, and positive (PLR) and negative likelihood ratios (NLR) were calculated with 95% confidence intervals. Complete prehospital and outcome data were available for 58 FAST-positive subjects among 158 subjects with prehospital suspicion for stroke/TIA. For NIHSS ≥ 15, C-STAT had a sensitivity of 77% (95% CI 46–95) and a specificity of 84% (95% CI 69–93). For NIHSS ≥10, the sensitivity of C-STAT was 64% (95% CI 41–83) and specificity 91% (95% CI 76–98). For presence of LVO, the sensitivity was 71% (95% CI 29–96) and specificity 70% (95% CI 55–83); and for overall CSC need sensitivity was 57% (95% CI 34– 78) and specificity was 79% (95% CI 61–91). In this pilot study, the C-STAT was easily performed in the prehospital setting by EMS providers without formalized training, is comparable to other published tools in test characteristics, and may inform appropriate CSC triage beyond LVO ascertainment alone in patients with suspected stroke in the prehospital setting. Larger prospective studies are warranted to advance prehospital stroke care.

**Meisel ZF, Mathew R, Wydro GC, Mechem C, Pollack CV, Katzer R, et al. Multicenter Validation of the Philadelphia EMS Admission Rule (PEAR) to Predict Hospital Admission in Adult Patients Using Out-of-hospital Data. Academic Emergency Medicine. 2009;16(6):519-25.**

The Philadelphia EMS Admission Rule (PEAR) was developed to discriminate between hospital admission and discharge for a general population of ED patients transported by EMS. The score consisted of six weighted elements that generated a total score (0–14): age ≥ 60 years (3 points); chest pain (3); shortness of breath (3); dizzy, weakness, or syncope (2); history of cancer (2); and history of diabetes (1). The objective of this study was to validate PEAR for predicting hospital admission using routinely collected out-of-hospital information.

The authors performed a multicenter retrospective cohort study of 1,500 randomly selected, adult patients transported to six separate emergency departments in Philadelphia (EDs; three community and three academic hospitals in three separate health systems) by a city-run emergency medical services (EMS) system over a 1-year period. A total of 1,102 patients met inclusion criteria. The admission rate for the entire cohort was 40%, and individual hospital admission rates ranged from 28% to 57%. Overall, 34% had a score of ≥4, and 29% had a score of ≥ 5. Area under the ROC curve (AUC) for the combined cohort was 0.83 for all admissions and 0.72 for intensive care unit (ICU) admissions; AUCs at individual hospitals ranged from 0.72 to 0.85. The admission rate for a score of ≥ 4 was 77%; for a score of ≥ 5 the admission rate was 80%. Use of the PEAR scoring system may help inform systems that seek to use directed diversion to match patients who are likely to need inpatient resources with hospitals that have adequate bed capacity. Implementation and impact analysis studies are needed before this rule can be broadly implemented.

**Newgard CD, Zive D, Holmes JF, Bulger EM, Staudenmayer K, Liao M, et al. A Multisite Assessment of the American College of Surgeons Committee on Trauma Field Triage Decision Scheme for Identifying Seriously Injured Children and Adults. Journal of the American College of Surgeons. 2011;213(6):709-21.**

The American College of Surgeons Committee on Trauma and the Centers for Disease Control jointly developed the National Trauma Triage Protocol: Field Triage Decision Scheme (FTDS) to identify trauma patients who would benefit from trauma center care. The FTDS is based on the stepwise identification of four aspects of clinical presentation involving physiologic criteria (PHY), anatomic criteria (ANA), mechanism of injury criteria, and special considerations criteria that are evaluated in a sequential fashion to identify patients who should be transported to a trauma center. Physiologic criteria focus on vital signs and Glasgow Coma Scale (GCS); anatomic criteria include specific injury patterns such as penetrating trauma, flail chest, crush injury, etc; mechanism of injury criteria focuses on high risk patterns such as falls from specific height, high speed vehicular crash, motorcycle accident etc; and special considerations include extremes of age, comorbidities, burns, pregnancy, anticoagulated status, etc.

The aim of this study was to evaluate the diagnostic performance of the FTDS for identifying major trauma patients (Injury Severity Score [ISS] ≥16) among a large and diverse population of injured patients evaluated by EMS providers across multiple sites. This was a retrospective cohort study of injured children and adults transported by 94 EMS agencies to 122 hospitals in 7 regions of the Western U.S. from 2006 through 2008. Patients who met any of the field trauma triage criteria (per EMS personnel) were considered triage positive. The majority of sites were urban and suburban, though some outlying rural areas are also included. All sites have established trauma systems with designated Level I/II trauma centers and standardized trauma triage protocols. The primary analysis to validate the ability of the FTDS was designed to identify patients with ISS ≥ 16, regardless of the type of hospital to which they were initially transported. Sensitivity was calculated as the proportion of seriously injured patients identified by field triage criteria and specificity as the proportion of non-seriously injured patients that did not meet triage criteria. Under-triage (the proportion of major trauma patients missed by the triage criteria) was calculated from 1 - sensitivity and over-triage (the proportion of minimally injured patients meeting triage criteria) as 1 – specificity.

A total of 122,345 injured patients were evaluated and transported by EMS over the 3-year period, of whom 34.5% met at least one triage criterion and 5.8% had ISS ≥ 16. The overall sensitivity and specificity of the criteria for identifying major trauma patients were 85.8% (95% CI 85.0 – 86.6%) and 68.7% (95% CI 68.4 – 68.9%). Triage sensitivity and specificity differed by age: 84.1% and 66.4% (0 – 17 years); 89.5% and 64.3% (18 – 54 years); and 79.9% and 75.4% (≥ 55 years). The sensitivity of the Field Triage Decision Scheme for identifying major trauma patients is lower and specificity higher than previously described, particularly among elders.

**Newton M, Tunn E, Moses I, Ratcliffe D, Mackway-Jones K. Clinical navigation for beginners: the clinical utility and safety of the Paramedic Pathfinder. Emergency Medicine Journal. 2014;31(e1):e29-34.**

Two general triage protocols named Pathfinders were developed, one for patients who had suffered trauma and the other for patients with medical conditions. The Pathfinders were derived from the Manchester Triage System to safely categorize patients into four groups (or pathways): those requiring emergency care, those requiring urgent care, those whose care could be safely delivered in the community with appropriate support and those who could self-care. The aim of the study was to evaluate the clinical utility and safety of these triage support tools (Pathfinders) using a mixed clinician sample.

Pathfinders were then applied by ambulance clinicians to 481 patients who had been transported to eight evaluation sites in England. Preferred (gold standard) patient dispositions were established by senior medical practitioners using both ambulance and ED clinical records. The clinical utility of ambulance clinicians using Pathfinders was evaluated against this gold standard. Both Pathfinders pre-exclude patients with Pre-Hospital Early Warning Score (PHEW) of >4.

The Medical Pathfinder was applied to 367 patients (76.3%) and the Trauma Pathfinder to 114 (23.7%). Agreement between ambulance clinician and gold standard was achieved in 387 cases (80.5%) giving the tools a combined sensitivity of 94.83% and specificity of 57.9%. A total of 20.9% of medical patients and 30.7% of trauma patients who had been transported to hospital could have been safely cared for elsewhere. Ambulance clinicians using Pathfinders have demonstrated acceptable levels of sensitivity in identifying patients who require ED care. The actual impact of the tools in clinical practice will be dependent on the provision of suitable alternatives to ED.

**Ng CJ, Chien CY, Seak JC, Tsai SL, Weng YM, Chaou CH, et al. Validation of the five-tier Taiwan Triage and Acuity Scale for prehospital use by Emergency Medical Technicians. Emergency Medicine Journal. 2019;36(8):472-8.**

The Taiwan Triage and Acuity Scale, implemented by the Taiwan Ministry of Health and Welfare in 2010, retains most of the features of the Canadian Triage and Acuity Scale (CTAS). It has been modified by dividing the chief complaint into a non-trauma category, comparable to the CTAS, and a trauma category with the chief complaint list organized by anatomical region and environmental injury. In addition, vital sign ranges for hemodynamic stability and pain severity ratings that omit chronic pain differ from the CTAS. The TTAS triage levels are categorized as follows: level 1, resuscitation; level 2, emergency; level 3, urgent; level 4, less urgent; and level 5, non-urgent. This study aimed to determine the inter-rater reliability of the five-level Taiwan Triage and Acuity Scale (TTAS) when used by emergency medical technicians (EMTs) and triage registered nurses (TRNs).

This was a prospective observational study in Taoyuan City, Taiwan, between 1 July and 31 December 2014. After training in five-level triage, EMTs triaged patients arriving to the ED and agreement with the nurse triage (TRN) was assessed. Subsequently, these trained research EMTs rode along on ambulance calls and assigned TTAS scores for each patient at the scene, while the on-duty EMTs applied their standard two-tier Taiwan prehospital triage scale (TPTS) and followed standard practice, blinded to the TTAS scores. 493 patients were assigned two-tier Taiwan Prehospital Triage System (TPTS) priority scores by working EMTs and TTAS scores by the research EMT. The accuracy of the TTAS scores in the field for prediction of hospitalization and medical resource consumption was analyzed using logistic regression and a linear model, respectively, and compared with the accuracy of the current two-tier prehospital triage scale.

Inter-rater agreement between EMTs and TRNs for triage of ED patients was very good (κw=0.825, CI 0.750 to 0.900). The hospitalisation rate was 73.9% and 20.1% for TPTS emergent and non-emergent level patients, respectively. Using TTAS, the hospitalisation rates for acuity levels I–V were 90.9%, 53.4%, 26.5%, 7.2% and 0.0%, respectively. For the outcome of hospitalization, TTAS five-level system (Akaike’s Information Criteria (AIC)=486, area under the curve (AUC)=0.75) showed better discrimination compared with TPTS two-level system (AIC=508, AUC=0.66). In conclusion, the five-level TTAS assignment by EMTs showed good inter-rater agreement with triage RNs in the hospital setting. The application of TTAS by EMTs at the scene proved to be more accurate in predicting the need for hospitalization and medical resources than the current two-level TPTS.

**Noorian AR, Sanossian N, Shkirkova K, Liebeskind DS, Eckstein M, Stratton SJ, et al. Los Angeles Motor Scale to Identify Large Vessel Occlusion: Prehospital Validation and Comparison With Other Screens. Stroke. 2018;49(3):565-72.**

Prehospital scales have been developed to identify patients with acute cerebral ischemia (ACI) due to large vessel occlusion (LVO) for direct routing to Comprehensive Stroke Centers (CSCs), but few have been validated in the prehospital setting, and their impact on routing of intracranial hemorrhage (ICH) patients has not been delineated. The LAMS is a 3-item (Facial Droop, Arm Drift, and Grip Strength), 0- to 10-point motor stroke-deficit scale, which had initially been developed from the Los Angeles Prehospital Stroke Screen (LAPSS), for the general purpose of characterizing stroke deficit severity in the field. The purpose of this study was to validate the Los Angeles Motor Scale (LAMS) for LVO and CSC-appropriate (LVO ACI and ICH patients) recognition and compare the LAMS to other scales. The performance of LAMS was assessed in identifying: 1) LVOs among all ACI patients, and 2) CSC-appropriate patients among all suspected strokes. Additionally, the LAMS administered post-arrival was compared concurrently with 6 other scales proposed for paramedic use and the full National Institutes of Health Stroke Scale (NIHSS).

Data were prospectively analyzed from consecutive patients transported to UCLA Medical Center in Los Angeles, California, USA, throughout the study period (2004–2012). Patients with likely stroke, indicated by a positive modified LAPSS, and within 2 hours of last known well, were enrolled in the trial and LAMS was administered prehospital by paramedics. Among 94 patients, final diagnoses were acute cerebral ischemia in 76% (due to LVO in 48% and non-LVO in 28%), ICH in 19%, and neurovascular mimic in 5%. The prehospital LAMS score cutoff at ≥ 4 showed good performance both in identifying LVOs among cerebral ischemia patients (sensitivity 0.76, specificity 0.65, and accuracy 0.72) and in identifying CSC-Appropriate patients among all suspected stroke transports (sensitivity 0.73, specificity 0.71, and accuracy 0.72). When concurrently performed in the Emergency Department post-arrival, the LAMS showed comparable or better accuracy versus the 7 comparator scales, for LVO among ACI (accuracies: LAMS 0.70, other scales 0.62–0.68) and CSC-appropriate case (accuracies: LAMS 0.73, other scales 0.56–0.73). The LAMS performed in the field by paramedics identifies large vessel occlusion and CSC-Appropriate patients with good accuracy. The LAMS performs comparably or better than more extended prehospital scales and the full NIHSS.

**Okuno Y, Yamagami H, Kataoka H, Tahara Y, Tonomura S, Tokunaga H, et al. Field Assessment of Critical Stroke by Emergency Services for Acute Delivery to a Comprehensive Stroke Center: FACE2AD. Translational Stroke Research. 2019;12:12.**

The FACE2AD scale was designed to predict large vessel occlusion. It assigns one point each for the presence of facial palsy, arm weakness, consciousness impairment, atrial fibrillation, and diastolic blood pressure ≤ 85 mmHg and assigning two points for the presence of eye deviation.

The aim of this study was to design a simple prehospital stroke scale for emergency medical service (EMS) paramedics to identify patients with LVO. The derivation cohort was extracted from the database of the National Cerebral and Cardiovascular Center, Osaka, between April 2012 and February 2015. 1157 out of 1419 eligible patients transferred to the hospital by the EMS because of suspected stroke within 24 h of onset were retrospectively examined.

The accuracy of this scale was then prospectively validated in 502 consecutive patients who were transferred to 4 stroke centers in Japan during a 5-month period. Its accuracy was compared with those of 4 previously reported scales. Logistic regression was used to examine how well the FACE2AD scale could predict the probability of LVO. Sensitivity and specificity measurements were calculated for LVO (+) to produce a receiver operating characteristic (ROC) curve and to determine the optimum FACE2AD scale threshold for the diagnosis of LVO.

The prevalence of LVO in the total cohort was 2% for score 0 or 1, 8% for score 2, 18% for score 3, 35% for score 4, 59% for score 5, 67% for score 6, and 76% for score 7. Conveniently enough, when the FACE2AD scale score was 5 or more, the prevalence of LVO (%) was estimated to be the 10-fold value of the score. Clearly, a FACE2AD scale score ≥ 5 was associated with a high likelihood of LVO (> 50%), whereas a score < 3 was associated with a low likelihood of LVO (< 10%). With the cutoff ≥ 3, the AUC was 0.87 (95% CI; 0.86–0.88) and the sensitivity, specificity, positive predictive value, and negative predictive value to predict LVO were 0.83, 0.78, 0.39, and 0.97, respectively. The FACE2AD scale is a simple tool to detect ischemic stroke patients with LVO by the EMS.

**Silcock DJ, Corfield AR, Gowens PA, Rooney KD. Validation of the National Early Warning Score in the prehospital setting. Resuscitation. 2015;89:31-5.**

The National Early Warning Score (NEWS) stratifies patients into risk categories based on observed heart rate, respiratory rate, systolic blood pressure, arterial oxygen saturation, temperature, and conscious level; plus an additional weighting if the patient receives oxygen therapy. Patients are then risk stratified based on the resulting aggregate score into low, medium, and high-risk groups. Patients with a low aggregate score but who score in the highest category for any single observation are classified as at least medium risk.

This study aimed to retrospectively evaluate the performance of the NEWS in identifying patients at risk of death or deterioration in the prehospital setting in Scotland. Details of all emergency ambulance crews dispatched with an intention to transfer to the Royal Alexandra Hospital (RAH) were obtained from the Scottish Ambulance Service data warehouse and collated along with information relating to hospital outcome over a two-month period between October 1st and November 30th, 2012. NEWS values for each patient encounter were calculated retrospectively from the clinical parameters obtained.

Studied outcomes included survival to discharge or 30 days, death within 48 hours of hospital admission, ICU admission within 48 hours of hospital admission, and a composite adverse outcome of death or ICU admission within 48 hours. 1684 patients were analysed. All three of the primary endpoints and the combined endpoint were associated with higher NEWS scores (p < 0.01 for each). The medium-risk NEWS group was associated with a statistically significant increase in ICU admission (RR = 2.466, 95% CI 1.0–6.09), but not in-hospital mortality relative to the low risk group. The high-risk NEWS group had significant increases in 48 h mortality (RR 35.32 [10.08–123.7]), 30 day mortality (RR 6.7 [3.79–11.88]), and ICU admission (5.43 [2.29–12.89]). Similar results were noted when trauma and non-trauma patients were analysed separately. Elevated NEWS among unselected prehospital patients is associated with a higher incidence of adverse outcomes. Calculation of prehospital NEWS may facilitate earlier recognition of deteriorating patients, early involvement of senior Emergency Department staff and appropriate critical care.

**Skjot-Arkil H, Pontoppidan LL, Laursen JO, Giebner M, Andersen JD, Mogensen CB. Do prehospital providers and emergency nurses agree on triage assignment?: an efficacy study. European Journal of Emergency Medicine. 2019;26(1):29-33**

The Danish Emergency Process Triage (DEPT) is a five-level triage system based on a combination of vital signs and a presenting complaint algorithm. DEPT categorizes the condition of the patient into five degrees: red (life threatening), orange (critical), yellow (stable, but potentially unstable), green (stable), and blue (unaffected). The color determines the urgency of medical attention required: red requires immediate action, whereas blue demands reevaluation every fourth hour. First, the triage level based on the measured vital signs is determined. Second, the triage based on the presenting complaint is determined. The presenting complaint algorithm consists of 50 main presenting complaints. The triage level based on vital signs and based on presenting complaints are then combined, and the final triage level is determined by the variable that indicates the highest degree of urgency. The aim of this study was to investigate the agreement on triage level between prehospital providers and emergency department (ED) nurses in clinical practice when using the DEPT triage system.

This prospective and observational efficacy study was performed between 1 April 2015 and 1 July 2015. The study involved 80 ED nurses at the urban Hospital of Southern Denmark, a level-2 trauma center, and 160 prehospital providers working in the hospital’s coverage area. Of the 724 paired data charts, 292 (40%) paired data charts were used correctly by both professionals. Patients were triaged by prehospital providers while being transported by ambulance to the ED, and by ED nurses upon arrival. The inter-rater agreement was determined using the κ [9] with 0.5 weights. The vital signs were categorized into triage levels according to the triage model. A two-sided probability of P value of less than 0.05 was considered statistically significant.

The triage agreement was measured only if DEPT was used correctly by both prehospital providers and ED nurses. DEPT was used correctly by both professions in 292 patients. In 182 (62%) patients the prehospital providers and the ED nurses agreed on the same triage level. This equals to κ = 0.47 [95% confidence interval (CI): 0.41–0.56]. When considering the triage based on vital signs the agreement was 72% (κ = 0.46; 95% CI: 0.41–0.47), and based on presenting complaint the agreement was 46% (κ = 0.41; 95% CI: 0.37–0.44). There was a moderate interrater agreement on triage assignment between ED nurses and prehospital providers. They agreed on final triage more often if they agreed on triage based on vital signs rather than presenting complaints.

**Smith DT, Snyder A, Hollen PJ, Anderson JG, Caterino JM. Analyzing the Usability of the 5-Level Canadian Triage and Acuity Scale By Paramedics in the Prehospital Environment. Journal of Emergency Nursing. 2015;41(6):489-95.**

The Emergency Severity Index (ESI) and the Canadian Triage and Acuity Scale (CTAS) are two 5-level triage instruments with similar reliability and validity findings. CTAS and ESI classify patient acuity on a scale from 1 to 5, with 1 being most urgent and 5 being least urgent. The ESI triage process uses an algorithm based on patient acuity and anticipated resource need to determine triage category. In contrast, CTAS uses an extensive list of clinical complaints, symptoms, and modifiers, at strategic times, to direct users toward a specific classification.

This study sought to determine if a relationship exists between the CTAS scores assigned by prehospital paramedics and the ESI score assigned by nurses in the emergency department, as well as to determine whether either instrument correlates with patient admission. Data analyses included descriptive statistics, χ2 statistics, and hierarchical regression analysis. This descriptive correlational study was conducted with the city of Columbus (Ohio) Division of Fire (CFD) and The Ohio State University (OSU).

The analysis included 2,222 patients. There was a poor relationship between the CTAS and the ESI at the facility (P = .599, κ = –0.003). The final regression model explained 32.9% of the admission variance (P b .001). The model correctly predicted 61.5% of admissions, with an 82% accuracy rate for all other forms of disposition and an overall model prediction rate of 73.7%. Using the CTAS, paramedics can predict admission comparably with nurses using the ESI. However, both instruments showed weakness in over- and under-triage rates. Additional studies are indicated to better understand prehospital paramedic triage and its impact on throughput.

**Sung S, Kang CY, Lee HY, Lee JH, Kim OH, Youk H, et al. Correlation between the pre-hospital triage scale and emergency department triage scale. Hong Kong Journal of Emergency Medicine. 2019;26(5):281-7.**

The Simple Triage and Rapid Treatment (START) triage system was classified by paramedics into five stages of patient triage: Emergent (E)—patients with at least one unstable vital sign, major symptoms of chest pain, unconsciousness, dyspnea, respiratory arrest, palpitations, cardiac arrest, paralysis, severe trauma patients and patients judged by paramedics to require prompt treatment within minutes; Less Emergent (LE)—if not Emergent but need treatment within a few hours; Potentially Emergent (PE)—all patients who are not Emergent, Less Emergent, but need emergency care; Extra: this is not an emergency transfer and for outpatient visits, reserved patients, and so on; and Death (D). This study aimed to analyze the correlation between the triage systems at the pre-hospital and in-hospital stages (START vs Korean Triage and Acuity Scale, KTAS) to better determine the priority of medical care for emergency patients and to utilize the results as basic data for the future development of a pre-hospital triage system.

This retrospective study was conducted with data obtained from a regional emergency medical center in Korea. A total of 1028 patients were included in the study from April to May 2016 out of 7141 patients who visited the center. The inter-rater agreement was used to analyze the correlation between the pre-hospital and in-hospital triage systems. The significance level was judged to be statistically significant when it was less than 0.05.

Upon reclassifying the pre-hospital and in-hospital triage systems into three levels, among the 289 patients (28.1%) in level 3 of the pre-hospital triage, 79 (27.3%) were reclassified as the highest level (Resuscitation) in the in-hospital triage. The kappa coefficient as a measure of agreement between the two triage systems was very low at 0.211 (95% confidence interval, 0.164–0.258), and the kappa coefficient of the paramedic category was 0.232 (95% confidence interval, 0.161–0.303). There is a low agreement between the pre-hospital and in-hospital triage systems. Therefore, there is a need to develop a more systematic and unambiguous pre-hospital triage system based on the KTAS.

**Suzuki J, Nakai N, Kondo N, Tsuji H, Inagaki R, Furukawa S, et al. Ten-Year Evaluation of the TOYOTA Prehospital Stroke Scale for Tissue Plasminogen Activator Intravenous Therapy in the Real World. Cerebrovascular Diseases. 2018;46(3-4):184-92**

The TOPSPIN prehospital stroke scale for tissue plasminogen therapy consists of 5 items each scored from 0 to 10 points: consciousness, atrial fibrillation, language disorder, hemiparesis of the upper extremities, and hemiparesis of the lower extremities. EMS crew recorded the TOPSPIN score at the time of contact with the patient. The aim of this study was to analyze the performance characteristics of TOPSPIN for stroke recognition 10 years after its implementation. Consecutive patients who were transferred to the hospital and evaluated by Toyota city ambulance services, which services approximately 420,000 people in the Aichi prefecture, central Japan, were prospectively enrolled using the TOPSPIN from December 2006 to January 2017.

The correlation between total TOPSPIN scores and NIHSS scores for patients with ischemic stroke was assessed using Spearman’s rank correlation test. Independent predictors of outcome were determined using multivariate logistic regression analysis by entering significant variables from the univariate analysis. A total of 1482 patients were enrolled; stroke was definitively diagnosed in 1,134 patients (76.5%), including 628 ischemic stroke, 34 TIA events, and 472 hemorrhagic stroke events; the remaining 348 (23.5%) patients did not have a stroke. Among 628 patients with ischemic stroke, 130 (20.7%) received intravenous recombinant t-PA treatment, endovascular therapy, or both. The PPV for EMS stroke recognition was 82.8% when the total TOPSPIN score was ≥3 points. A moderate correlation was observed between the total TOPSPIN score and the NIHSS score in patients with ischemic stroke (r2 = 0.53, p < 0.001). The presence of atrial fibrillation, older age, lower blood pressure, and lower total TOPSPIN score was more commonly associated with ischemic stroke than with hemorrhagic stroke. In multivariable logistic regression analysis, the presence of atrial fibrillation was independently associated with ischemic stroke (OR 2.33; 95% CI 1.61–3.40). The TOPSPIN is a simple prehospital stroke scale that includes an assessment of atrial fibrillation. Detection of atrial fibrillation in the prehospital stage may point to a higher probability of ischemic stroke.

**Taqi MA, Sodhi A, Suriya SS, Quadri SA, Farooqui M, Salvucci AA, et al. Design, Application and Infield Validation of a Pre-Hospital Emergent Large Vessel Occlusion Screening Tool: Ventura Emergent Large Vessel Occlusion Score. Journal of Stroke and Cerebrovascular Diseases. 2019;28(3):728-34.**

The Ventura Emergent LVO Scale (VES) comprises 4 components: eye deviation, aphasia, neglect, and obtundation with score range 0-4. The score of greater than or equal to 1 is considered as LVO positive. A positive VES along with positive Cincinnati scale prompts ELVO activation.

The performance characteristics of VES were evaluated retrospectively by collecting unidentified data from the electronic medical charts from a single center and EMS agency in Ventura County, Los Angeles, California, from 2016 to 2017. A total 184 patients were included in the final analysis. Of 62 (33.7%) patients who were called VES positive from the field, 36 (58%) were diagnosed with LVO. The mean NIHSS on arrival was 16 in VES positive and 5 in VES negative patients. VES had 94.7% sensitivity, 82.4% specificity, with 58.1% PPV and 98.4% NPV. It showed 84.9% accuracy. VES is an effective and simplified prehospital screening tool for detection of LVO in the field.

**Tsai LH, Huang CH, Su YC, Weng YM, Chaou CH, Li WC, et al. Comparison of prehospital triage and five-level triage system at the emergency department. Emergency Medicine Journal. 2017;34(11):720-5.**

This study aimed to compare the two-level Taiwan Prehospital Triage System (TPTS) with the five-level Taiwan Triage and Acuity Scale (TTAS) at ED arrival regarding the prediction of patient outcomes and the utilization of medical resources.

In the prehospital phase, patients were triaged using the two-level TPTS. The criteria for TPTS emergent level patients are those who present with abnormal vital signs, including a GCS score <14, or high or low blood sugar via finger stick, or critical chief complaints suggestive of acute stroke, ischemic heart disease, seizures, life-threatening intoxication, precipitate labor, snake bite, or drowning. In trauma events, criteria for emergent level of acuity include the presence of a specific physiological or anatomical injury or the evidence of a high-risk mechanism of injury, in addition to abnormal vital signs. All other patients were considered non-emergent.

This was a retrospective cohort study of adult patients transported via EMS in northern Taiwan in 2012. In order to compare the predictability of hospitalization rate between these two triage systems, the sensitivity, specificity, positive predictive value (PPV), negative predictive value (NPV), positive likelihood ratio (LR+), negative likelihood ratio (LR−) in TPTS (emergent vs non-emergent) and TTAS (levels 1–2 vs 3–5) were calculated. Among 4430 enrolled patients, 25.2% and 74.8% were classified as emergent and non-emergent by TPTS; 44.1% and 55.9% were classified as levels 1–2 and levels 3–5 by TTAS. Of the TPTS emergent patients, 15.2% were classified as TTAS levels 3–5, whereas 30.4% of TPTS non-emergent transports were classified as TTAS levels 1–2 at the ED. TTAS levels 1–2 better predicted hospitalization rate than TPTS emergent level with a sensitivity of 70.3% (95%CI 68.3% to 72.2%) versus 41.1% (95% CI 39.0% to 43.2%), and a negative predictive value of 74.8% (95%CI 73.4% to 76.0%) versus 62.6% (95% CI 61.7% to 63.5%). This study concludes the current prehospital triage system is insufficient and inappropriate in classifying patients transported to the ED. The present study offers supporting evidence for the introduction of a five-level triage system to prehospital EMS systems.

**Vaclavik D, Bar M, Klecka L, Holes D, Cabal M, Mikulik R. Prehospital stroke scale (FAST PLUS Test) predicts patients with intracranial large vessel occlusion. Brain and Behavior. 2018;8(9):7.**

The FAST PLUS test has two parts. The first part is the FAST test, which is employed in all possible cases of stroke occurrence. This test consists of the following items: Facial palsy, any failure of Arm motor function, and Speech. The FAST test is considered positive if the score is at least one. The second part of the FAST PLUS test evaluates only the presence of severe arm or leg motor deficit. The FAST PLUS test results are considered positive when there is a positive general FAST test score and severe paresis of a leg or an arm or both.

The aim of this study was to determine the performance characteristics of the FAST PLUS test as administered by paramedics for predicting LVO confirmed by CT angiography (CTA). This is a prospective observational cohort study of prehospital patients with suspected stroke (FAST test positive) who were transported by emergency medical services to one of the three stroke centers in Ostrava, Czech Republic, according to their territory.

The study included 435 patients. LVO were found in 124 patients (28%). Sensitivity was 93%, specificity was 47%, PPV was 41%, NPV was 94%, and AUC was 0.65 for LVO. Intracerebral hemorrhage (ICH) was identified in 48 patients (11%). It was found that the FAST PLUS test had a high sensitivity for LVO stroke.

**van Laarhoven JJ, Lansink KW, van Heijl M, Lichtveld RA, Leenen LP. Accuracy of the field triage protocol in selecting severely injured patients after high energy trauma. Injury. 2014;45(5):869-73.**

In the Netherlands, the Dutch field triage protocol is utilized for major trauma triage. The parameters in the field triage protocol can be categorized into three groups: physiological condition (P), mechanism of trauma (M) and injury type (I). The aim of the present study was to evaluate the diagnostic accuracy of the current field triage protocol in high-energy trauma patients. This was a prospective cohort study of all high-energy trauma patients from 2008 to 2011 in the region Central Netherlands. 1607 patients were included of which 13.8% had major trauma, defined as ISS>=16. Sensitivity and specificity of the field triage protocol was 89.1% (95%CI 84.4–92.6) and 60.5% (95% CI 57.9–63.1), respectively. Positive predictive value was 26.5% (95%CI 23.4-29.8) and negative predictive value was 97.2% (95CI 95.9-98.1). The undertriage rate was 10.9% (95%CI 7.4–15.7) and the overtriage rate was 39.5% (95%CI 36.9–42.1). The overall rate of under-triage (10.8%) was mainly influenced by a high rate of undertriage in the group of patients with only a positive mechanism criterion.

**Voskens FJ, van Rein EAJ, van der Sluijs R, Houwert RM, Lichtveld RA, Verleisdonk EJ, et al. Accuracy of Prehospital Triage in Selecting Severely Injured Trauma Patients. JAMA Surgery. 2018;153(4):322-7.**

In the Netherlands, allocation of trauma patients to the appropriate level of trauma care is guided by the Dutch Field Triage Protocol made for EMS professionals. This protocol is based on the CDC/ASCOT Field Triage Decision Scheme. The aim of the study was to prospectively evaluate the ability of the Dutch field triage system to identify severely injured adult trauma patients. Undertriage was defined as the proportion of severely injured patients (Injury Severity Score, ISS ≥16) erroneously transported to level II or III hospitals. Overtriage was defined as the proportion of patients with an ISS of less than 16 transported to a level I trauma center.

Prehospital and hospital data of all adult trauma patients during 2012 to 2014 transported with the highest priority by emergency medical services professionals to 10 hospitals in Central Netherlands were prospectively collected. A total of 4950 trauma patients were evaluated of which 436 (8.8%) patients were severely injured. The under-triage rate based on actual destination facility was 21.6% (95% CI, 18.0-25.7) with an over-triage rate of 30.6% (95% CI, 29.3-32.0). Analysis of the protocol itself, regardless of destination facility, resulted in an under-triage of 63.8% (95% CI, 59.2-68.1) and over-triage of 7.4% (95% CI, 6.7-8.2). The compliance to the field triage trauma protocol was 73% for patients with a level 1 indication. The undertriage rate in elderly patients is high at 38.6% (95% CI, 30.8-47.2). A high-energy trauma mechanism resulted in an undertriage rate of 9.1% (95% CI, 5.8-14.2). More than 20% of the patients with severe injuries were not transported to a level I trauma center. These patients are at risk for preventable morbidity and mortality. This finding indicates the need for improvement of the prehospital triage protocol.

**Zhao H, Coote S, Pesavento L, Churilov L, Dewey HM, Davis SM, et al. Large Vessel Occlusion Scales Increase Delivery to Endovascular Centers Without Excessive Harm From Misclassifications. Stroke. 2017;48(3):568-73.**

The Rapid Arterial Occlusion Evaluation (RACE), Los Angeles Motor Scale (LAMS), Cincinnati Prehospital Stroke Severity Scale (CPSSS), Field Assessment Stroke Triage for Emergency Destination (FAST-ED), and Prehospital Acute Stroke Severity scale (PASS) are all recently published scales, using simplifications of items from the National Institutes of Health Stroke Scale (NIHSS) to optimize prediction of LVO.

The aim of this study was to examine the diagnostic performance of published LVO triage scales in a representative Australian cohort of suspected acute stroke, with a specific emphasis on scale performance in atypical clinical presentations. The prehospital stroke scale values were derived from the baseline National Institutes of Health Stroke Scale (NIHSS) scored by doctors and analyzed for diagnostic performance compared with imaging. Prospective data were collected from consecutive ambulance-initiated stroke alerts at 2 stroke centers, with patients stratified into typical (LVO with predefined severe syndrome and non-LVO without) or atypical presentations (opposite situations).

Of a total of 565 patients, atypical presentations occurred in 31 LVO (38% of LVO) and 50 non-LVO cases (10%). The negative predictive values were similar for all scales (91%–93%), but there was a trend to higher positive predictive value for RACE, LAMS, and FAST-ED (all with PPV 48%). Most scales correctly identified >95% of typical presentations but <20% of atypical presentations. Misclassification attributable to atypical presentations would have resulted in 4 M1/internal carotid artery occlusions, with NIHSS score ≥6 (5% of LVO) being missed and 9 non-LVO infarcts (5%) bypassing the nearest thrombolysis center. Atypical presentations accounted for the bulk of scale misclassifications, but the majority of these misclassifications were not detrimental, and use of LVO scales would significantly increase timely delivery to endovascular centers, with only a small proportion of non-LVO infarcts bypassing the nearest thrombolysis center.

**Zhao H, Pesavento L, Coote S, Rodrigues E, Salvaris P, Smith K, et al. Ambulance Clinical Triage for Acute Stroke Treatment: Paramedic Triage Algorithm for Large Vessel Occlusion. Stroke. 2018;49(4):945-51.**

A 3-step ambulance clinical triage for acute stroke treatment (ACT-FAST) was created to improve specificity by recognizing only severe clinical syndromes and optimizing paramedic usability and reliability. It was designed by identifying clinical deficits associated with a high predictive value for LVO using the Royal Melbourne Hospital stroke database. The ACT-FAST algorithm consists of (1) unilateral arm drift to stretcher <10 seconds, (2) severe language deficit (if right arm is weak) or gaze deviation/hemineglect assessed by simple shoulder tap test (if left arm is weak), and (3) eligibility and stroke mimic screen.

The study objective was a retrospective and prospective validation the ACT-FAST LVO identification algorithm. In retrospective (n=565) and prospective paramedic (n=104) validation, ACT-FAST displayed higher overall accuracy and specificity, when compared with existing LVO triage scales. Agreement of ACT-FAST between paramedics and doctors was excellent (κ=0.91; 95% confidence interval, 0.79–1.0). The full ACT-FAST algorithm assessed by paramedics on 60 patients showed high overall accuracy (91.7%), sensitivity (85.7%), specificity (93.5%), positive predictive value (80%) and negative predictive value (95.6%) for recognition of endovascular-eligible LVO.
